# Not all transmembrane helices are born equal: Towards the extension of the sequence homology concept to membrane proteins

**DOI:** 10.1186/1745-6150-6-57

**Published:** 2011-10-25

**Authors:** Wing-Cheong Wong, Sebastian Maurer-Stroh, Frank Eisenhaber

**Affiliations:** 1 Bioinformatics Institute (BII), Agency for Science, Technology and Research (A*STAR), 30 Biopolis Street, #07-01, Matrix, 138671 Singapore; 2 School of Biological Sciences (SBS), Nanyang Technological University (NTU), 60 Nanyang Drive, 637551 Singapore; 3 Department of Biological Sciences (DBS), National University of Singapore (NUS), 8 Medical Drive, 117597 Singapore; 4 School of Computer Engineering (SCE), Nanyang Technological University (NTU), 50 Nanyang Drive, 637553 Singapore

## Abstract

**Background**

Sequence homology considerations widely used to transfer functional annotation to uncharacterized protein sequences require special precautions in the case of non-globular sequence segments including membrane-spanning stretches composed of non-polar residues. Simple, quantitative criteria are desirable for identifying transmembrane helices (TMs) that must be included into or should be excluded from start sequence segments in similarity searches aimed at finding distant homologues.

**Results**

We found that there are two types of TMs in membrane-associated proteins. On the one hand, there are so-called simple TMs with elevated hydrophobicity, low sequence complexity and extraordinary enrichment in long aliphatic residues. They merely serve as membrane-anchoring device. In contrast, so-called complex TMs have lower hydrophobicity, higher sequence complexity and some functional residues. These TMs have additional roles besides membrane anchoring such as intra-membrane complex formation, ligand binding or a catalytic role. Simple and complex TMs can occur both in single- and multi-membrane-spanning proteins essentially in any type of topology. Whereas simple TMs have the potential to confuse searches for sequence homologues and to generate unrelated hits with seemingly convincing statistical significance, complex TMs contain essential evolutionary information.

**Conclusion**

For extending the homology concept onto membrane proteins, we provide a necessary quantitative criterion to distinguish simple TMs (and a sufficient criterion for complex TMs) in query sequences prior to their usage in homology searches based on assessment of hydrophobicity and sequence complexity of the TM sequence segments.

**Reviewers**

This article was reviewed by Shamil Sunyaev, L. Aravind and Arcady Mushegian.

## Background

In a previous publication [1], we provided evidence that the inclusion of transmembrane segments (TMs) or signal peptides (SPs) into models of protein domains can result in apparently statistically significant, yet unrelated hits when these models are applied to similarity searches in protein sequence databases. This observation can be rationalized within the current formulation of the sequence homology concept [1-6]: SP/TM regions are essentially hydrophobic stretches that, in a first approximation, are similar to each other regardless of evolutionary origin due to functional pressures. The alignment with a SP/TM region produces many coincidences of hydrophobic positions that create the appearance of hydrophobic pattern similarity, otherwise the key criterion for similarity of the hydrophobic core and of the fold among globular proteins [2]. Whereas for globular domains, sequence similarity is interpreted as evolutionary divergence from a common ancestor, these sequence similarities are more likely the result of evolutionary convergence.

More generally, SPs/TMs can be considered a special case of non-globular segments in protein sequences where the concept of sequence homology originally developed for soluble globular domains is not directly applicable [1,2]. Non-globular segments are characterized by amino acid compositional bias or primitive repetitive patterns [2,7,8]. Sequence similarity among non-globular segments is not so much an evidence for common evolutionary ancestry as for the similarity of functionally critical physico-chemical constraints. Therefore, it is strongly advisable to exclude non-globular sequence segments from starting sequences used for similarity searches in sequence databases [2,4,8-11].

The work presented in this article has essentially emerged in response to an important comment by one of the reviewers of our previous publications [1]; namely, the complete exclusion of TMs from domain models in libraries such as Pfam [12,13] "would be a huge disservice to the community" and certain domain models involving TM regions have proven instrumental in protein family classification as, for example, in the cataloging of membrane transporters by Saier and coworkers [14-16]. Indeed, with additional expert insights and manual interference into the annotation process [17,18], certain types of multi-membrane proteins can be satisfactorily defined or classified via sequence similarity. Some representative examples are the 4-TM helices domain HTTM (horizontally transferred transmembrane) [19] in the SMART database [20,21], the rhomboid protein family [22-24], the TCDB (Transporter classification database) [14,15] and the GPCRDB (G-protein coupled receptor database) [25,26].

In [1], we mention that SP/TM regions have a trend towards low sequence complexity compared with α-helices from structural proteins; yet, in multi-TM proteins, this trend is not as pronounced as in TMs of single-TM proteins or as in signal peptides. In this work, we build on this observation and derive criteria that distinguish TM regions with structural/functional particularities (to be called 'complex' TMs) from mere hydrophobic stretches (to be called 'simple' TMs). In one type of integral membrane proteins, the general architecture consists of a globular segment that confers its biological function and a single helix that anchors the protein to the lipid bilayer as a result of physiological requirements (it may merely look like a hydrophobic stretch, most likely due to convergent evolution). An example is the APMAP (adipocyte plasma membrane-associated protein) which is a Type II membrane protein with a N-terminal anchor and a C-terminal six-bladed β-propeller extracellular domain with potential hydrolase activity [27]. The other type is exemplified by multi-spanning TM proteins where several TM helices are stitched together via rather short loop regions. With minimal soluble globular content if at all, their biological functions must be governed by the TM helices. As an example, the function of rhodophosin, a 7-TM GPCR (G-protein coupled receptor) is conferred by six of its seven TM helices [28-30]. From a probabilistic perspective, it would be an extremely rare event for six TM helices to evolve independently through convergent evolution to confer the protein's enzymatic function. Homology (due to common ancestry) would be the more viable explanation for such "functional" TMs. Considerable problems for interpretation arise for the 'in-betweeners', that is, when a protein is mostly globular; yet, it has a few TM helices the functional role of which is not clear. An example is the PIG-P protein (phosphatidylinositol N-acetyl-glucosaminyl transferase subunit P) [31]. 40% of the sequence is covered by its two TM helices and 60% is globular.

For sequence similarity applications within the sequence homology concept (i.e., for the extension of the homology concept to membrane proteins), a quantitative criterion for distinction between complex and simple TMs would be very helpful, not only in the context of automated annotation pipelines. In this work, we explore the empirical distribution of TM regions collected from the protein sequence database with regard to sequence properties and we find fundamental evidence supporting the classification in complex and simple TMs. Simple TMs were found to have less variable amino acid composition and to be enriched with hydrophobic residues while the complex ones harbor polar/charged residues and glycine/proline residues that change structural parameters of TM helices. As a consequence of their increased hydrophobicity [32,33], the simple helices tend to have a higher propensity for membrane insertion than the complex helices. Although we find that simple TMs prevail in single TM proteins, they can also co-exist with complex helices in multi-TM proteins. Using the TCDB dataset [14,15], we show that masking of simple TM helices in homology searches improves the false-discovery rate of searches without compromising on the sensitivity while masking the complex ones wreaked havoc to the latter.

Likewise, complex helices can also be observed in proteins with only a few or a single TM region. We show that the application of the sequence homology concept can be justified for such complex helices that we find in single-TM-spanning proteins implicated in diseases and in immune signaling [34]. Taken together, either simple or complex TM helices can exist in integral membrane proteins regardless of their topologies. In this regard, common ancestry appears not an exclusive business of the complex TM helices within the multi-spanning membrane proteins.

We present a quantitative necessary criterion based on hydrophobicity and sequence complexity assessment of TM regions to distinguish simple TMs from other cases. We also explore the likelihood of occurrence of simple or complex TMs depending on the number of TMs in the membrane protein. For the convenience of the reader, all datasets used in this work, many raw computation results as well as a PERL program for TM classification (calculation of hydrophobicity, sequence complexity and z-score of TM regions) are available at the WWW-site http://mendel.bii.a-star.edu.sg/SEQUENCES/ProblemDomains-TM-classification/ for download.

## Results

### Peculiarities in the sequence property distributions of integral membrane proteins suggest that TM helices can be simple or complex

In total, 181132 TM helices from single and multi-spanning membrane proteins were extracted from the UniProt annotation file (dated 16-09-2010) based on the keyword FT_TRANSMEM. For each TM helix, we calculated two values - hydrophobicity and sequence complexity. For the hydrophobicity computation, the octanol-to-interface scale [35] is used as a measure of membrane insertion propensity (although with reversed sign to have high values coincide with high hydrophobicity). The window size of 19 is taken from the original work [36], the final value is the average over all windows. For determining sequence complexity, we used a modified form of Shannon's entropy formula where IVL is considered as a single group and all other amino acid residue types as individual (see below for justification). Window size is 12 and we average over all windows of a given TM.

**Figure 1:**
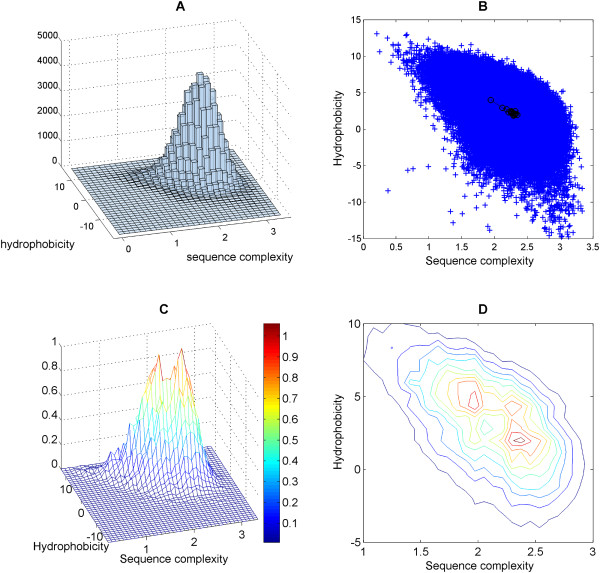
**Distribution of UniProt-derived TM segments in the complexity/hydrophobicity plot**. 181132 transmembrane helices from single and multi-spanning membrane proteins were extracted based on the keyword FT_TRANSMEM described in the UniProt annotation file (dated 16-09-2010). The three-dimensional histogram (Figure 1A) shows a fin-shaped distribution across the diagonals of the complexity and hydrophobicity space. The cross-section of the histogram (Figure 1B) shows a tear-shaped pattern. Furthermore, the medians of 15 sets of spanning membrane proteins (containing 1 to 15 TM helices respectively) were computed and denoted by the black circles. The medians were connected in ascending order, starting from the single-spanning set. The trace of the complexity and hydrophobicity from the single-spanning set to the four-TM spanning set indicates a progressive shift towards high complexity and low hydrophobicity. The medians converge to almost a singular cloud beyond the five-transmembrane-spanning set. The three-dimensional surface plot (Figure 1C) shows the hybrid distributions of the single-spanning and those with at least five membrane-spanning TM helices. The single-spanning ones appear more 'simple' while, as a trend, the others seem more 'complex'. The two to four-spanning helices are excluded as they were considered to be 'in-betweeners'. Two distinct peaks (in red) can be seen in the surface plot, denoting 'simple' and 'complex' populations respectively. The contour plot (Figure 1D) emphasizes on the two peaks.

Figure 1A shows the histogram of all TM helices in the sequence complexity/hydrophobicity space. The cross-section of this skewed histogram in Figure 1B. The medians of 16 sets of membrane-spanning proteins (15 sets each containing TMs from 1-, 2-, 3-, ...,14- or 15-TM proteins and another of TMs from proteins with 16 or more TMs) are denoted by black circles and connected in an ascending order starting from the single-TM-spanning set. The connected path clearly shows that the medians move towards high complexity/low hydrophobicity and finally converge to a singular cloud beyond the 5-TM spanning set. This observation suggests that, on average, the single-TM-spanning and the multi-TM-spanning transmembrane helices are different in sequence complexity and hydrophobicity.

To amplify this observation, the 'in-betweener' sets (2TMs, 3TMs and 4TMs) were removed. The three-dimensional surface plot (Figure 1C) shows the overlay of two distributions, of the single-TM-spanning sets and of the combined set of TMs from proteins with at least 5 TM helices. Consequently, two peaks (in red) can be observed. The cross-section of this surface plot (Figure 1D) emphasizes on these two peaks. The right peak corresponds to a location on the singular cloud of multi-spanning medians in the scatter plot while the left peak represents the single-TM-spanning set. This presentation supports the conclusion that, essentially, there are two types of TM helices in the space of complexity and hydrophobicity. Those with low sequence complexity and higher hydrophobicity will be called 'simple' TMs; we define those with high sequence complexity and lower hydrophobicity as 'complex' TMs.

**Figure 2:**
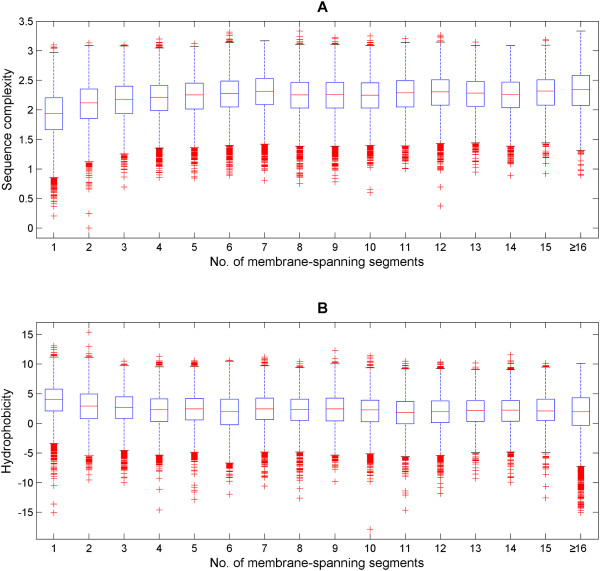
**Trends of complexity and hydrophobicity as a function of the number of TMs per protein in UniProt**. The boxplots of complexity (Figure 2A) and hydrophobicity (Figure 2B) of the single-spanning to 15-transmembrane and beyond spanning sets are shown. The medians of the complexity and hydrophobicity measures increase with the number of spanning TM helices found in each set. Interestingly, the complexity boxplots highlighted an excess of outliers on the lower part of the complexity axis (suggestive of low-complexity sequence segments) while the hydrophobicity boxplots emphasized the excess of outliers on the bottom part of the hydrophobicity axis (towards charged composition). This is independent of the size of the membrane-spanning proteins. Taken together, this is somewhat contrary to the expectation that TM helices are simple and purely hydrophobic. Therefore, it raises the notion that some TM helices are 'simple' and others are 'complex' regardless of the number of TMs in a protein.

Furthermore, the distributions of the complexity and hydrophobicity of the 16 membrane spanning sets are examined individually (Figure 2). The medians of the complexity measure increases while the hydrophobicity decreases with the number of membrane-spanning helices and stabilize beyond the 5-TM-spanning set. The spreads across the data sets seem consistent. Perhaps, the most intriguing observation is the simultaneous enrichment of low-complexity outliers in the complexity boxplots (Figure 2A) and low-hydrophobicity outliers in the hydrophobicity boxplots (Figure 2B). This is independent of the number of membrane-spanning helices. This is particularly interesting for the single-TM-spanning set where there is the expected, clear trend of membrane anchors being low in complexity and high in hydrophobicity. The data shows that there are a sizeable number of outliers not following the trends and we can reconcile this seemingly contradictory observation by acknowledging that simple and complex TMs can occur in proteins regardless of the total number of TMs.

### Enrichment of hydrophobic residues in TM anchors and charged/structural residues in functional TM helices and their respective propensity for membrane insertion

Here, we wish to examine whether specific amino acid compositional bias can divorce the TM helices into the proposed classes of simple and complex in nature. For this purpose, we analyze the following sets of TM helices (see Methods for detail): (i) 1767 signal anchors (with "Signal-anchor" as part of FT_TRANSMEM annotation in UniProt), (ii) 303 membrane anchors (all others with "Anchor" annotation), (iii) 1741 functional TM helices from UniProt (additional FT_METAL, FT_BINDING or FT_ACTIVE record for a position covered with FT_TRANSMEM), (iv) 83 functional TMs from the previous set reappearing in SCOP membrane class [37,38]. Note that any substrings and repeated sequences of these datasets were removed via the cd-hit algorithm [39] (with options -n 5 -c 1) prior to this study.

Although UniProt's strategy to label TMs as anchors is not fully transparent, we use these sets to detect trends likely representative of simple TMs since we might assume that simple TMs are enriched among the anchors. The other two sets are thought to be examples for and be enriched in complex helices. Additionally, the membrane and signal anchors were treated as two distinct classes of transmembrane anchors instead of one due to the considerable difference in sample size that might otherwise affect the results. The SCOP-derived set (iv) is an especially well curated subset of UniProt-derived set (iii); we use both to detect possible bias in the results that might arose from sampling bias in the small dataset and by possible annotation errors in the large one. Thus, the concordance of results between the datasets as observed below certifies that possible ambiguities caused by different evidence levels in the UniProt-derived sets (for example, by data with labels such as "POTENTIAL" or "BY SIMILARITY" are not relevant for the conclusions with regard to the summary statistics.

For each of the four sets, we determined the amino acid composition. That leaves us with 4 rows of 20 amino acid counts for analysis. For the pairwise comparison of two rows, we apply the proportion test (via 20-by-2 contingency table [40]) at a significance level of P = 0.05 (corresponding to χ^2 ^= 30.14). We find statistical difference at χ^2 ^= 521 for the SCOP-derived functional TM set and χ^2 ^= 1209 for the UniProt-derived set when tested against the membrane anchors set. Similarly, the results were significant at χ^2 ^= 786 and χ^2 ^= 3737 for the SCOP-derived and UniProt-derived functional TM set respectively in tests against the signal anchors. Together, these initial results imply that there is a general difference in amino acid composition between the simple and complex TM helices.

The investigation was furthered by detecting the specific residues that are enriched in the simple and complex TM helices. For this purpose, each set was characterized by twenty two-value rows (the count of occurrence of a specific amino acid type and all other occurrences). Like before, the same set-to-set comparisons were made. For each case, twenty 2-by-2 contingency tables formulated as a binomial comparative trial at a family-wise error rate of 0.05 (significance level of 0.0025 per test) were used (see chapter 23, pp.491-500 of ref. [40]). The two-sided test was selected since no prior assumption of the directions of the proportions was made. Each set-to-set comparison would yield a pair of enriched residues; one from the anchors and another from the functional TM-helices.

Based on results (see Additional file 1, Supplementary Tables 1), the comparisons against the functional TM-helices (SCOP-derived and UniProt-derived) elucidated IVLC and IVLCK as the enriched residues from membrane anchors (1A and 1B) while this was LVC and LVCKST for the signal anchors (1C and 1D). On the other hand, the same comparisons also found the highly similar sets of residues: RDEHNFGP enriched in either functional TM helix set with respect to the membrane anchor set (1A and 1B) and RDEHNMFWGP enrichment in the functional TMs relative to the signal anchor set (1C and 1D). Essentially, these findings imply that the anchors are enriched with aliphatic hydrophobic residues (LVI) while the functional TM-helices are enriched with charged (RDEH), structurally important (GP) and aromatic (FW) residues at their generally hydrophobic background. Given the consistencies in the results, the effects of both sampling bias in the small dataset (SCOP-derived) and the possible annotation errors in the large one (UniProt-derived) are likely to be minimal.

**Table 1:** Test of difference between the summary statistics of functional TM-helix sets (SCOP-derived versus UniProt-derived)

Section	Quantitative Criteria	Windows size	SCOP-derived			UniProt-derived			
			
			***μ***_***c***_	***σ***_***c***_	***n***_***c***_	***μ***_***c***_	***σ***_***c***_	***n***_***c***_	p-value
A	Sequence complexity (based on IVL group)	10	2.31	0.28	83	2.28	0.29	1741	0.32
		12	2.42	0.29	83	2.40	0.30	1741	0.56
		15	2.56	0.31	83	2.53	0.32	1741	0.39
		18	2.68	0.31	83	2.63	0.32	1741	0.15

B	Hydrophobicity scale	19	0.41	2.91	83	0.64	2.85	1741	0.48

The summary statistics of the quantitative criteria (sequence complexity/hydrophobicity; column 2) for the SCOP-derived and UniProt-derived functional TM-helix sets are given in section A and B of Table 1 respectively (column 4-6, 7-9). The summary statistic (*μ*_*c*_, *σ*_*c*_, *n*_*c*_) denotes the mean, standard deviation and sample size for the sequence complexity measures for a given window size (10,12,15 and 18; column 3) while the summary statistic (*μ*_Φ_, *σ*_Φ_, *n*_Φ_) denotes the mean, standard deviation and sample size for the hydrophobicity measures for the window size of 19. The p-values (last column) are computed from the two-tailed t-test. None of the tests return a significant result which means that the summary statistics between the SCOP-derived and UniProt-derived functional TM-helix sets are similar. As such, calculations of false-negative rates based on either functional TM-helix sets should give similar conclusions.

Our observation in complex TM helices of enrichment with charged and structural residues and the association with specific function beyond membrane anchoring is compatible with findings from independent laboratories. Arginine (R) mutations within a TM helix of the Tar receptor can drive homodimer dissociation and heterodimer association [41]. Polar/charged residues (QNED) in TMs drive strong helix associations [42]. The GXXXG motif in a TM of the human erythrocyte protein glycophorin A and bacteriophage M13 major coat protein is necessary for TM-TM association [43,44]. Proline residues are generally implicated in the folding/assembly [45] and structural stability of TM helices [44,46-48].

White *et al*. have experimentally determined the propensity of membrane insertion for each amino acid residue in terms of an octanol-to-interface scale [35]. In summary, they concluded that aromatic and hydrophobic residues have high propensity for insertion while the charged and structural residues sit on the other extreme [32,33]. Remarkably, our statistically derived enriched residues are well correlated to these experimentally derived hydrophobicity scales. The Pearson's coefficients for the four set-to-set comparisons ranges from 0.36 to 0.56 (see Additional file 2, Supplementary Tables 2). As a guide, a correlation coefficient of greater than 0.5 is large, 0.5 to 0.3 is moderate, 0.3 to 0.1 is small while anything smaller than 0.1 is trivial [49]. Taken together, this establishes the notion that anchors being mainly hydrophobic have a higher propensity for membrane insertion than the functional TM-helices since the latter are enriched with charged and structural residues. Indeed, this conclusion forms the basis of distinction between the simple and complex TM helices in nature and, therefore, justifies for their separation.

**Table 2:** False-positive and false-negative rates of membrane/signal anchors versus SCOP/UniProt-derived functional TM-helix sets

Section	Description of comparisons	window size	f = 0.84		f = 1.0		f = 1.282		f = 1.645		f = 1.98	
			
			FPR	FNR	FPR	FNR	FPR	FNR	FPR	FNR	FPR	FNR
A	membrane anchors versus functional TMs (SCOP-derived) based on IVL group	10	15.51	24.10	18.15	16.87	25.41	9.64	36.96	2.41	48.84	0.00
		12	14.85	21.69	18.15	16.87	24.75	9.64	38.28	2.41	47.52	1.20
		15	15.84	20.48	18.81	14.46	26.73	9.64	39.93	3.61	49.50	1.20
		18	16.50	18.07	19.47	15.66	29.04	10.84	40.92	3.61	48.84	1.20

B	membrane anchors versus functional TMs (UniProt-derived) based on IVL group	10	16.17	25.04	19.80	19.13	27.06	9.82	41.91	3.96	51.49	1.44
		12	17.16	24.30	19.80	18.84	27.39	9.65	42.57	3.73	51.49	1.49
		15	18.15	23.55	21.45	19.13	29.04	9.94	42.57	4.19	51.49	1.67
		18	19.14	23.26	22.11	19.18	33.99	10.45	44.55	4.19	53.14	1.72

C	signal anchors versus functional TMs (SCOP-derived) based on IVL group	10	23.71	24.10	31.01	16.87	43.52	9.64	59.59	2.41	72.16	0.00
		12	23.77	21.69	30.39	16.87	44.60	9.64	59.54	2.41	72.33	1.20
		15	22.52	20.48	30.56	14.46	43.92	9.64	60.05	3.61	72.72	1.20
		18	22.24	18.07	29.71	15.66	42.39	10.84	58.18	3.61	70.51	1.20

D	signal anchors versus functional TMs (UniProt-derived) based on IVL group	10	27.39	25.04	35.77	19.13	48.10	9.82	63.89	3.96	76.23	1.44
		12	26.26	24.30	34.47	18.84	47.71	9.65	63.38	3.73	75.66	1.49
		15	26.49	23.55	35.37	19.13	47.82	9.94	63.89	4.19	76.85	1.67
		18	28.35	23.26	35.99	19.18	48.84	10.45	64.86	4.19	77.42	1.72

E	membrane anchors versus functional TMs (SCOP-derived)	10	14.52	22.89	18.81	16.87	30.03	9.64	44.22	3.61	54.79	1.21
		12	15.51	20.48	19.14	14.46	30.36	10.84	44.55	4.82	55.12	2.41
		15	15.51	20.48	20.13	13.25	32.34	9.64	45.55	4.82	57.10	2.41
		18	16.17	18.07	20.79	14.46	33.00	10.84	44.88	4.82	57.10	1.21

F	membrane anchors versus functional TMs (UniProt-derived)	10	14.52	25.39	19.14	20.68	30.36	11.26	44.55	4.71	54.13	1.67
		12	14.85	25.16	18.81	20.74	31.35	11.55	43.23	4.42	54.13	1.55
		15	15.84	25.22	19.80	21.02	32.01	12.00	45.21	3.79	56.77	1.49
		18	18.15	24.87	23.10	20.97	34.98	11.20	48.19	4.19	58.42	1.67

G	signal anchors versus functional TMs (SCOP-derived)	10	25.24	22.89	32.26	16.87	46.07	9.64	62.93	3.62	75.21	1.21
		12	24.90	20.48	32.15	14.46	45.95	10.84	62.99	4.82	75.04	2.41
		15	24.39	20.48	31.64	13.25	45.84	9.64	63.50	4.82	74.99	2.41
		18	24.17	18.07	31.18	14.46	44.71	10.84	61.57	4.82	74.42	1.21

H	signal anchors versus functional TMs (UniProt-derived)	10	26.77	25.39	33.16	20.68	46.86	11.26	62.88	4.71	74.36	1.67
		12	25.24	25.16	32.54	20.74	45.73	11.55	61.74	4.42	73.91	1.55
		15	25.18	25.22	32.54	21.02	45.95	12.01	63.10	3.79	74.02	1.49
		18	26.83	24.87	33.73	20.97	48.22	11.20	64.35	4.19	76.51	1.67

Table 2 (section A-D) shows the data generated with collapse of aliphatic hydrophobic residues IVL into one residue group (to suppress sequence variability just among hydrophobic residues). Table 2 (section E-H) presents the data without collapse of IVL into one group for comparison. The error rates of the membrane and signal anchors versus the functional TM-helix sets are given in sections A & B (E & G) and in sections C & D (G & H) respectively. Column 1 gives the subsections (A to H) of the table. Column 2 describes the comparison of the respective datasets. Column 3 gives the window size of the sequence complexity measure. The window size for the hydrophobicity measure was fixed at 19. Column pairs (4,5), (6,7), (8,9), (10,11) and (12,13) give the false-positive rates (FPR) and false-negative rates (FNR) for the z-score thresholds at f = 0.84, 1.0, 1.282, 1.645 and 1.98 (corresponding to the theoretical false-negative rates of 20%, 16%, 10%, 5% and 2.5%) respectively.

### Quantitative criteria for the distinction of simple and complex TM helices

In the previous section, the existence of simple and complex TM helices and, hence, their separability was justified based on their amino acid compositional bias and residues' propensity for membrane insertion. Here, the hydrophobicity and the sequence complexity measures are proposed as the criteria to partition simple and complex TM helices.

As in the case of Figure 1, we characterize each TM segment with two values *x*_Φ _and *x*_*c*_. The hydrophobicity *x*_Φ _of a sequence segment is computed as average over membrane propensities for a moving sequence window (of size 19) using the sign-reversed octanol-to-interface scale [35,36]. For the complexity measure *x*_*c *_of a sequence segment, we average the Shannon's entropies calculated over the amino acid composition for a moving sequence window. For each entropy value, IVL is considered a single group while all other residues are dealt with individually (see methods). The choice of collapsing IVL into one group is based on the enrichment in aliphatic hydrophobic residues found in membrane and signal anchors (see previous section). For finding the optimum averaging window size for the complexity measure, we permuted across the values 10, 12, 15 and 18 (see below).

For the formulation of a criterion to distinguish simple and complex TMs, a z-score is introduced as an univariate measure (a sum of squares) of normalized complexity and hydrophobicity

(1) $$z\left(x_{\Phi },x_{c}\right)={\left(-1\right)}^{s}\left[\frac{{\left(x_{\Phi }-\mu_{\Phi }\right)}^{2}}{\sigma_{\Phi }^{2}}+\frac{{\left(x_{c}-\mu_{c}\right)}^{2}}{\sigma_{c}^{2}}\right]$$

where *μ *and *σ *are calculated as the mean and standard deviation values of all sequence segments in a given set of TM regions. The regression line between normalized hydrophobicity and complexity is read as

(2) $$\frac{x_{\Phi }-\mu_{\Phi }}{\sigma_{\Phi }}=\rho_{c,\Phi }\frac{x_{c}-\mu_{c}}{\sigma_{c}}$$

Note that *ρ*_*c*,Φ _is the correlation between sequence complexity and hydrophobicity. To determine the sign of the z-score in (1), we use the normal to the regressed line as decision criterion; i.e., the exponent *s *is set equal to one for

(3) $$\frac{x_{\Phi }-\mu_{\Phi }}{\sigma_{\Phi }}\geq -\frac{1}{\rho_{c,\Phi }}\frac{x_{c}-\mu_{c}}{\sigma_{c}}$$

and equal to zero otherwise. In this notation, TMs with high hydrophobicity and low complexity will have low z-scores (see methods for the derivation of the regressed line and normal equation).

**Figure 3:**
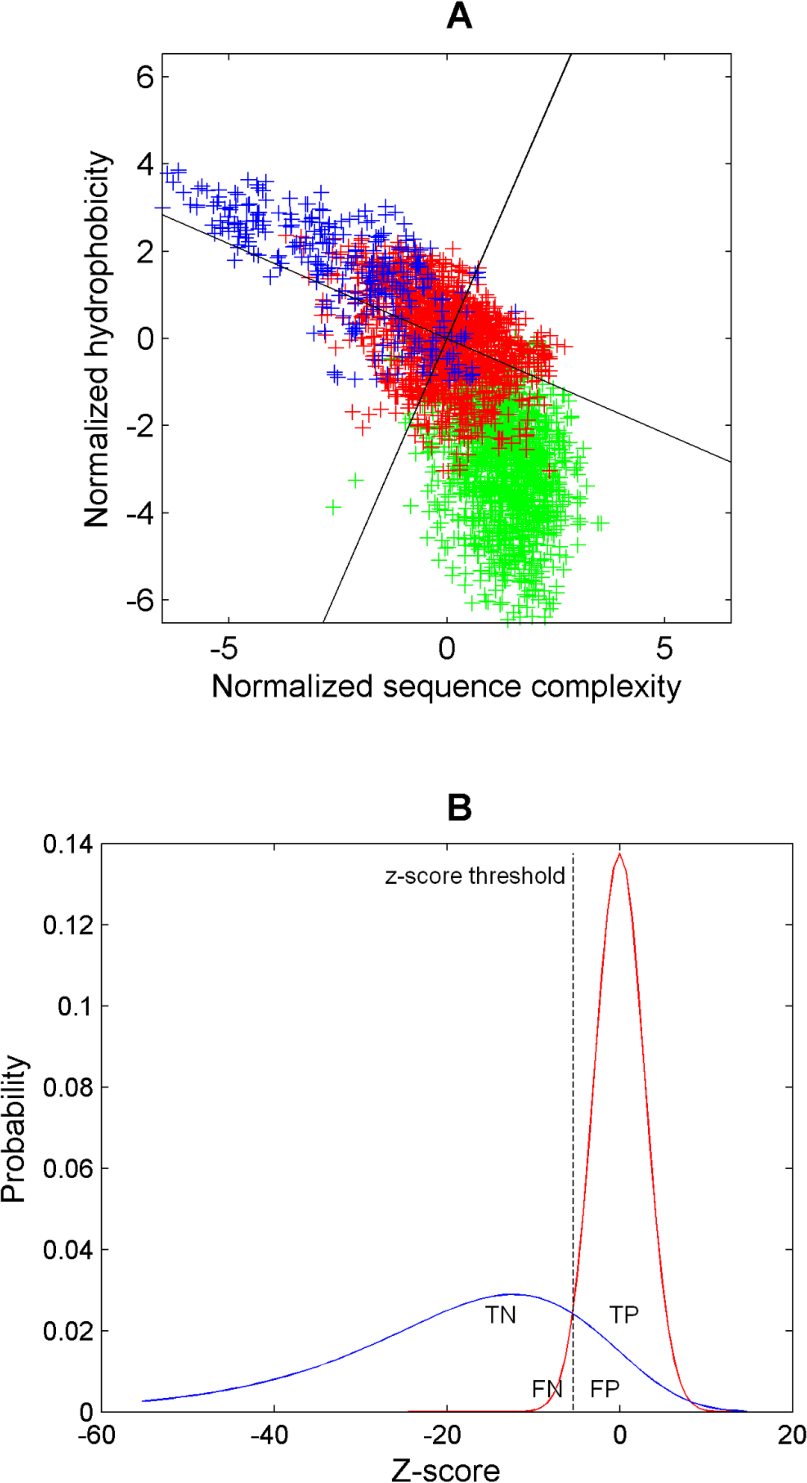
**Definition of the z-score with regard to the set of functional TMs**. Figure 3A shows the normalized sequence complexity/hydrophobicity measures of the membrane anchors (in blue), functional TM-helices (UniProt-derived; in red) and functional α-helices (in green). The univariate z-score is given by the squared sums of two normalized measures. The expectation of the z-scores for the functional TM-helices set is zero. The regression line (*ρ *= -0.436) for the functional TM set and its normal are indicated. The negative halve of the functional TM helices' z-scores is given by the inequality (3) while z-scores failing to satisfy the inequality makes up the positive halve. It is apparent that the majority of the membrane anchors have negative z-scores while the functional α-helices have positive z-scores. Figure 3B depicts the distributions of the membrane anchors (in blue) as simple TM helices and the functional TM-helices (in red) as complex TM helices. For illustrative purpose, the membrane anchors are approximated by the Gumbel distribution given its long left tail while the functional TM-helices are fitted to the normal distribution. For a given z-score threshold, the complex TM helices are partitioned into true-positives (TP) and false-negatives (FN) while the simple helices are divided into the true negatives (TN) and false-positives (FP). Consequently, the false-positive and false-negative rates can be determined for a given z-score threshold defined by equation (4).

In Figure 3A, we show how the membrane anchor TM set and the SCOP functional TM set relate to each other in the normalized hydrophobicity/complexity diagram. Thus, the comparison between two such sets can be used to derive a quantitative criterion in the hydrophobicity/complexity space by minimizing prediction errors. It should be noted that the sets of functional TMs (especially the one derived from SCOP) are much better defined than the sets of membrane or signal anchors; thus, a criterion for distinction between the two sets is more straightforwardly determined with the distribution of functional TMs. We found that the summary statistics of the SCOP- and of the UniProt-derived functional TM sets do not show any significant difference (Table 1). Therefore, we consider z-scores *z*(*x*_Φ_, *x*_*c*_) of any query TM relative to the summary statistics of the much larger UniProt-derived functional TM set (*μ*_Φ _= 0.64, *σ*_Φ _= 2.85, *μ*_*c *_= 2.4, *σ*_*c *_= 0.30, *ρ*_*c,Φ *_= -0.436; see 1). For illustration purposes in Figure 3B, we show the resulting approximated distribution of z-scores of membrane anchors and the UniProt-derived functional TMs. Essentially, the z-score can now be used to assess for a query TM whether it is simple (z-score < threshold) or complex (z-score ≥ threshold). Once a z-score threshold is fixed, the prediction process partitions the complex TM-helices into true-positives (TP) and false-negatives (FN) while the simple helices are divided into the true negatives (TN) and false-positives (FP).

To quantify the separability between the simple and complex TM helices via a z-score threshold, the error rates (false-positive and false-negative rates; see methods) were established for the set-to-set comparisons of the membrane and signal anchors against the functional TM-helices datasets (both SCOP-derived and UniProt-derived; see Table 1 for their summary statistics). With reference to the functional TM-helices sets, various z-score thresholds

(4) $$z_{threshold}\left(\mu_{\Phi }+f\sigma_{\Phi },\mu_{c}-f\sigma_{c}\right)$$

are defined at f = 0.840, 1.000, 1.282, 1.645 and 1.980 (equivalent to theoretical false-negative rates of 20%, 16%, 10%, 5% and 2.5% respectively for the one-tailed z-test) on the two measures since one does not know *a priori *the most appropriate threshold between simple and complex TM helices. Note that substituting expression (4) into (1) resolves into the equation *z*_*threshold*_(*μ*_Φ _+ *fσ*_Φ_, *μ*_*c *_-*fσ*_*c*_) = -2*f*^2^. Hence, the corresponding z-score thresholds are -1.41, -2.00, -3.29, -5.41 and -7.84 at f = 0.840, 1.000, 1.282, 1.645 and 1.980 respectively.

Table 2 (sections A and B) summarizes the false-positive and false-negative rates for the SCOP-derived and UniProt-derived functional TM-helices against the membrane anchors respectively while Table 2 (sections C and D) gives the error rates of the functional TM-helix sets against the signal anchors. Firstly, the data supports that the z-score of the functional TM sets is close to be normally distributed: For all f, the theoretical false-negative rates are very similar to the computed ones (the absolute difference is between 0.5% and 5%; see Table 2) and, as a trend, the computed rates are smaller than the theoretical ones especially for the larger f (1.645 and 1.980). This means that the z-scores of the functional TM-helices satisfy the normality assumption reasonably well for the range of thresholds studied, although the tails of the empirical distribution extend slightly more than in the theoretical normal distribution. But most importantly, the histograms of the functional TM-helices showed general symmetry (as an example, see Figure 5A). In contrast, a skewed distribution is sometimes suggestive of multiple distributions or populations (see chapter 2 in [50]). Together, this suggests that the functional TM-helices form a symmetrical unimodal distribution that is likely to contain itself as the only population.

With regard to the false-positive rates, there is only minimal difference across the various window sizes used to calculate sequence complexity. In Table 2 (sections A and B for the membrane anchors and sections C and D for the signal anchors), the absolute difference is within 7%. This suggests that window size used for sequence complexity computation has relatively small impact on the separability of complex and simple TM helices. The overall concordance is somewhat expected since the summary statistics of both complexity and hydrophobicity measures between the SCOP-derived and UniProt-derived functional TM-helices datasets were found to be statistically insignificant (Table 1). However, the average false-positive rates between the membrane and signal anchors show that the two sets are markedly different. Quantitatively speaking, the signal anchors have almost 20% more non-simple TM instances than the membrane anchors (absolute difference between the false-positive rates in Table 2; compare sections A/B and C/D). For the sake of completeness, we provide also the prediction rates computed without collapse of IVL into one group for sequence complexity calculations (see Table 2, sections E-H). The conclusions remain unchanged; yet, we think that, for future evaluation of TM helix properties, sequence complexity computations that are unaffected by variations just among the aliphatic hydrophobic residues are more appropriate since, then, the sequence complexity criterion becomes more orthogonal relative to hydrophobicity.

Finally, given the slightly preferential performance with changing complexity measure window size, the size of 12 is used throughout the rest of the work for the sake of simplicity.

### Relationships between TM helices, segments of low complexity, signal peptides & functional α-helices in globular domains and the peculiarities in the supposed simple TM populations of membrane and signal anchors

**Figure 4:**
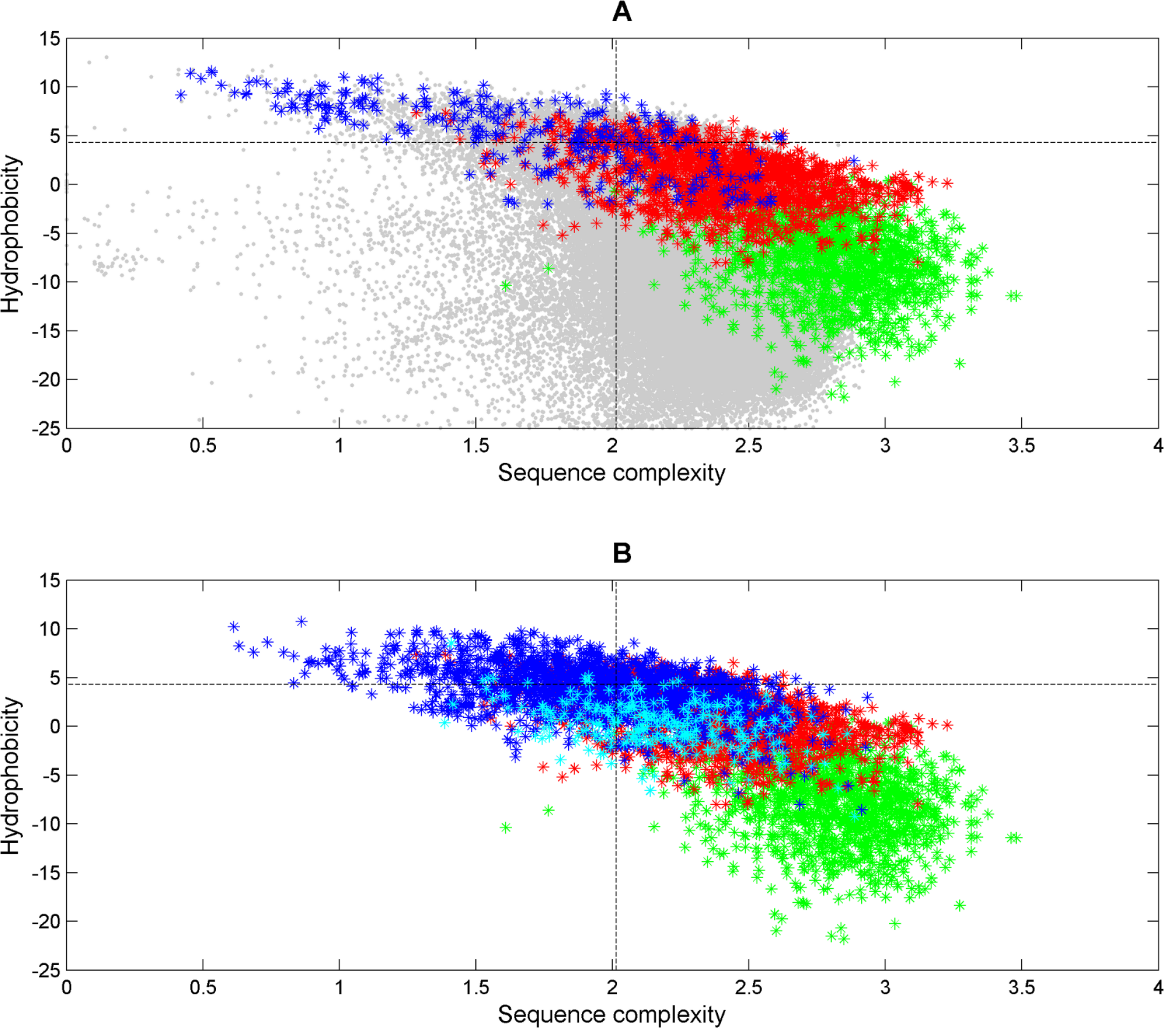
**Sequence complexity/hydrophobicity plot for membrane and signal anchors against functional TM-helices**. Figure 4A shows the sequence complexity/hydrophobicity plot of the membrane anchors (blue) against the functional α-helices (green), functional TM-helices (UniProt-derived; red) and low-complexity segments (SEG25/3.0/3.3-detected; gray). The dotted lines are defined by *x*_Φ _= *μ*_Φ _+ 1.282*σ*_Φ _and *x*_*c *_= *μ*_*c *_-1.282*σ*_*c *_where (*μ*_Φ_,*σ*_Φ_)and (*μ*_*c*_, *σ*_*c*_) are the summary statistic calculated from the UniProt-derived functional TM-helix set. This corresponds to a false-negative rate of 10% (at f = 1.282). Based on the plot, there is some overlap between the functional α- and TM-helices which justifies for the extension of the sequence homology concept for these cases. 55.8% (169 out of the 303) of the membrane anchors occupy the upper left quadrant, suggestive of simple TM helices. Figure 4B shows the complexity and hydrophobicity plot of signal anchors (blue) against the functional α-helices (green), functional TM-helices (UniProt-derived; red) and signal peptides (cyan). Similarly, the dotted lines define the boundaries at the false-negative rate of 10%. 30.1% of the signal anchors (531 out of the 1767) occupy the upper left quadrant, suggestive of simple TM helices.

Figure 4A shows the plot of complexity and hydrophobicity measures for membrane anchors against the functional TM-helices (UniProt-derived), functional α-helices and low-complexity sequences (SEG-detected; SEG25/3.0/3.3) [10]. The plot is partitioned into four quadrants by the two error margins defined by *x*_Φ _= *μ*_Φ _+ 1.282*σ*_Φ _and *x*_*c *_= *μ*_*c *_- 1.282*σ*_*c *_(f = 1.282 at 10% theoretical false negative rate) where (*μ*_Φ_,*σ*_Φ_) and (*μ*_*c*_, *σ*_*c*_) are the summary statistic calculated from the UniProt-derived functional TM-helix set.

The lower-right quadrant exhibits most instances of both the functional α-helices from globular proteins (1327 of 1330, 99.8%) and of the functional, i.e., complex TMs (1446 of 1741, 83.1%). Both groups have in common a trend towards high complexity/low hydrophobicity; yet, the trend is more pronounced for the α-helices. In the intermediate region between the two helix populations, there is considerable overlap. Some of these α-helices appear to belong to the group of δ-helices [51] that might function also as TM helices. The overlap between the populations can be seen as a justification to apply and, thus, to extend the sequence homology concept from the α-helices in globular domains to complex TMs.

In the other extreme, the upper-left quadrant (low complexity, very high hydrophobicity) contains 55.8% of the membrane anchor set (169 out of 303) that are examples of simple and more hydrophobic TM helices. Some overlap between the functional TM-helices and the membrane anchors is observed. Meanwhile, the overlap between the anchors and the low-complexity segments is much more pronounced. This justifies for the masking of these simple TM helices prior to sequence homology searches. However, the SEG algorithm [10] does not sample deep into the low complexity/very high hydrophobicity space. Instead, it samples almost only the low hydrophobicity space (97.9% or 465139 of 475207 instances of grey dots are below the hydrophobicity of -4.29 in Figure 4A) and also extends into the high complexity space of the functional TM and α-helices. As a consequence, the lone application SEG is insufficient to distinguish between the simple and complex TM helices.

Figure 4B shows the complexity/hydrophobicity plot of the signal anchors versus the functional TM-helices (UniProt-derived), functional α-helices and signal peptides (from proteins with structures in SCOP with the N-terminus missing, see Methods). Compared to the membrane anchors, the extent of overlap between the signal anchors and functional TM-helices is more pronounced. Only 30.1% (531 out of 1767) of the signal anchors occupy the upper-left, low complexity/very high hydrophobicity quadrant. As close analogues to the signal anchors, the signal peptides span a similar complexity space but approximately half the hydrophobicity space of the signal anchors. As a trend, the signal peptides are more hydrophilic. This is not a surprise since signal peptides have a canonical structure including polar, typically positively charged N-terminal tip followed by a quite uniformly hydrophobic region and a region of small residues, the latter involving the cleavage site [52]. In similarity searches, it is mainly the hydrophobic segment that causes false homologies [1]. Since the signal peptide cannot be part of the mature protein and, thus, of any of its globular domains, it is advisable to suppress signal peptides in protein domains in all cases of homology searches aimed at remnant sequence similarity levels.

As a matter of fact, the definition of signal anchors in UniProt is muddled. If we check the annotated signal anchors with the SignalP algorithm [52], 372 of the 1767 signal anchors were predicted as signal peptides both by the Hidden Markov Model (HMM) and the neural network versions of SignalP. Further 1057 of the signal anchors are predicted by either version of SignalP. Another 324 examples have been rejected as possible signal peptides by both versions. Out of them, 218 are N-terminal signal anchors (within the first 100 residues, see Methods), 106 are more C-terminally located. Despite of all these reservations, we can say that membrane anchor sequence segments, as a trend, are more hydrophobic/less complex than signal anchors and the trend is even stronger in the comparison with signal peptides.

As it is, the sequence sets of functional α-helices of globular proteins, signal peptides and low-complexity segments are better defined than the TM helices. For example, if all membrane and signal anchors are considered simple TMs, then 21% (62 out of 303) of the membrane anchors and 36% (638 out of 1767) of the signal anchors are in fact ill-defined based on the lower/right (high complexity/low hydrophobicity) quadrant, contrary to their UniProt annotations. To clarify the inter-relationships among the TM populations, the z-scores of each TM population are calculated using the summary statistics computed from the UniProt-derived functional TM-helices as a common reference (see Table 1 for the appropriate values of *μ*_Φ_,*σ*_Φ_, *μ*_*c*_, *σ*_*c*_). Most importantly, if the anchor sets are identical to the functional TM-helix set, their histograms should be similar to the latter. Otherwise, any peculiarities in the anchor distributions should appear.

**Figure 5:**
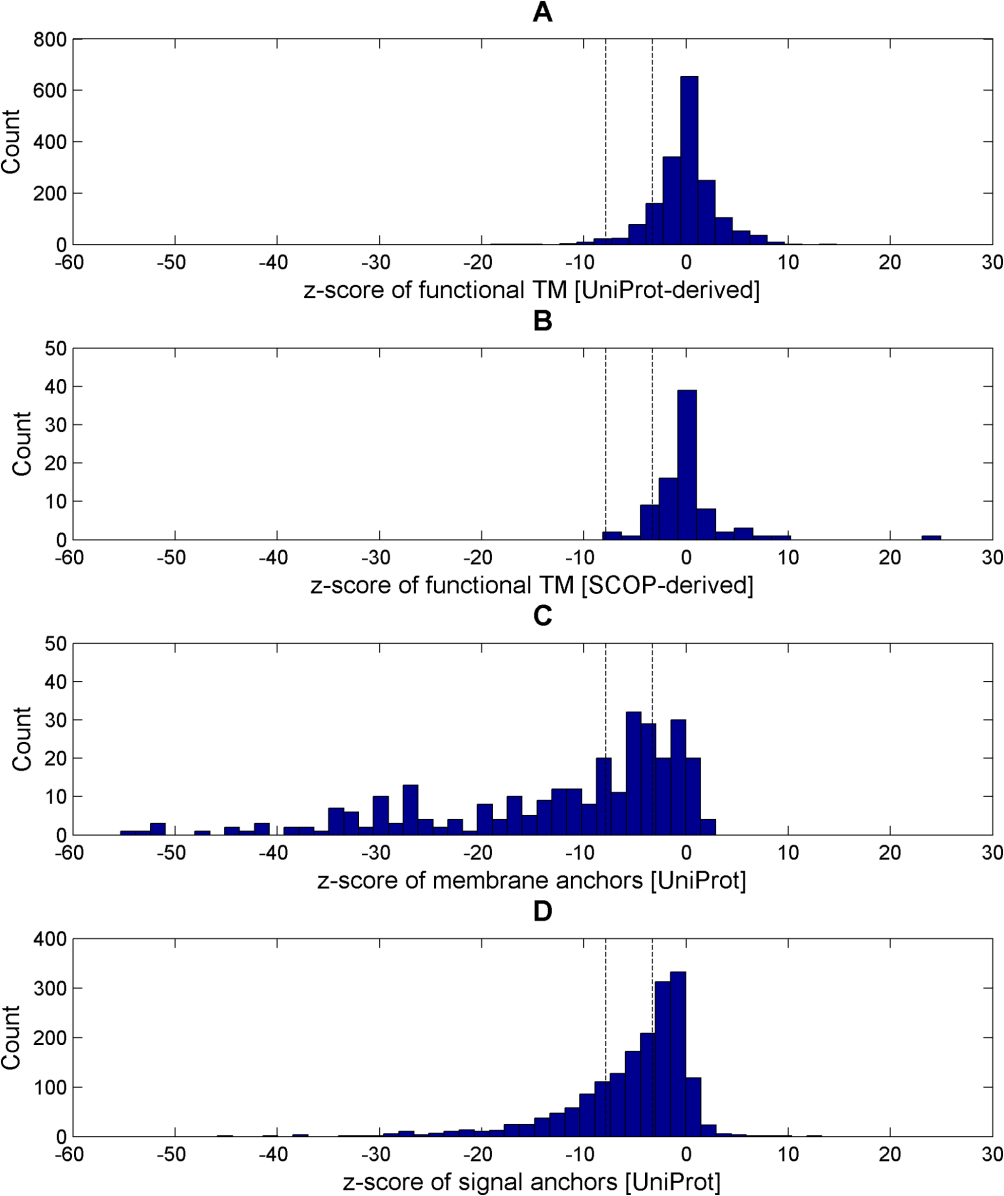
**Z-score histograms of the functional α-helices, functional TM-helices (SCOP/UniProt-derived), membrane anchors and signal anchors**. The z-score histograms for the functional TM-helices (SCOP-derived; Figure 5A and UniProt-derived; Figure 5B), membrane anchors (Figure 5C) and signal anchors (Figure 5D) are shown. The functional TM-helix sets have a characteristics long-tailed, symmetrical and unimodal shape histogram. On the other hand, the histograms of the membrane and signal anchor sets have a left-skewed long-tailed non-unimodality shape. This is suggestive of multiple populations. The two vertical dotted lines define a twilight zone between the functional TM-helices and the anchors based on the z-score thresholds of -7.84 and -3.29 that are calculated from f = 1.980 and f = 1.282 respectively via equation (4).

Figure 5 shows the histograms of the TM-helix populations: the functional TM-helices (SCOP-derived and UniProt-derived), membrane anchors and signal anchors in their respective z-scores. Unsurprisingly, both functional TM-helix sets display very comparable histograms but most notably, the histograms are both long-tailed, symmetrical and unimodal (see Figure 5A and 5B). In contrast, the anchor histograms are markedly different from that of the functional TM-helices (see Figure 5C and 5D). Their histograms are apparently left-skewed and long-tailed in nature. In the case of membrane anchors, non-unimodality can be observed explicitly (an apparent additional maximum at lower z-score around -30 in Figure 5C). In the z-score metric, 73% (220 out of 330) of the membrane anchors and 52% (924 out of 1767) of the signal anchors are considered as simple respectively at f = 1.282 (i.e. false-negative rate of 10%).

A plausible explanation for the skewness and non-unimodality observed in the anchors histograms is the existence of a mixed population of simple and complex TM helices. Firstly, this is compatible with our initial observation that a TM helix can be simple or complex. Secondly, our earlier enrichment analysis for residues in the simple and complex TM helices finds that the charged residue lysine (K) is found to be enriched in the anchor TM sets (see Table one of reference [34]). This is incompatible with the general observation that charged residues (RDEH) are characteristics of the complex ones. It is possible that some of these simple helices harboring lysine are in fact complex. If this is indeed the case, then complex TM helices (implying common ancestry) do also exist in single-spanning membrane proteins, contrary to their role as only anchors (implying convergent evolution). In other words, it means that TM helices in single-spanning membrane proteins may arise either through common ancestry or convergent evolution. As collateral, the general high false-positive rates in Table 2 of the anchors are likely exaggerated by the superimposed subset of complex TM helices.

### Complex TM helices in single-spanning TM proteins and their role in conferring homology

In the preceding section, the skewness and non-unimodality of the anchor histograms have hinted the alternate view that a complex TM helix can exist in the single-spanning membrane protein. On a separate note, case studies of single-spanning membrane proteins implicated in diseases and immune-signaling have shown that the TM helices can possess structural and functional roles well beyond serving as mere anchors. These helices are capable of protein complex assembly and signal transduction, very much like the small modular domains of the globular proteins [34]. To extend the sequence homology concept for TM helices in single-spanning membrane proteins, it is necessary for the complex helices to embody ancestry information while the simple ones do not.

We studied the 48 cases of functionally-related TM helices in Tables 1 and 2 of reference [34]. First, the full length sequences were searched against the SwissProt (downloaded on 26-12-2010) with BLASTP [9] to find the orthologues in various species of the respective proteins (to make use of SwissProt IDs for automated processing of search outputs). All other hits were ignored. For 27 out of the 48 cases, this search resulted in at least two hits and one of them is the full-length seed orthologue. Next, the same searches for the TM segments alone were executed. We used an E-value of 1000 to increase the sensitivity of detection for short sequences. The raw sequence comparison results are available additional files associated with this article.

For each of the 27 cases, the search results of the full length and TM-only segment were compared. Given that proteins can either be homologous or non-homologous to one another, only the following two extreme scenarios of the comparisons are meaningful; (i) Either the two result sets have complete matches exactly (ii) or the TM-only result set contains the TM segment of the seed orthologue itself as the sole hit.

The comparisons are tabulated in Table 3. Among the total of 27 valid cases, we find 19 cases of complete matches and 8 cases of sole hits. For 19 cases of complete matches, the TM sequences have demonstrated the capability of retrieving their orthologous helices. Their corresponding z-scores (calculated with summary statistic from the UniProt-derived functional TM set; see Table 1) range from -9.18 to -0.10. Meanwhile, the other 8 cases of sole hits failed to retrieve their orthologues. This is indicative of a discontinuity in ancestry information. Their z-scores are between -28.63 to -3.52. Overall, this gives rise to a conflict zone for the z-score between the 19 exact matches and 8 sole hits that spans from -9.18 to -3.52. Guided by this result, a twilight zone between simple and complex TM helices can be defined for a range of -7.84 to -3.29 that is bracketed by the z-score threshold of f = 1.980 (false-negative rate of 2.5%) and f = 1.282 (false-negative rate of 10%).

**Table 3:** 27 cases of functionally-related TM helices in single-spanning membrane proteins

Section	Description of match	Gene name	Seed UniProtID	***x***_***c***_	***x***_***φ***_	z-score	Orthologues of seed
A	Cases of 19 complete matches between full length and TM-only sequence	FGFR2	FGFR2_HUMAN	2.39	1.26	-0.10	FGFR2_CHICK;FGFR2_MOUSE; FGFR2_NOTVI;FGFR2_PLEWA; FGFR2_DANRE;FGFR2_XENLA
		ectodysplasin	EDA_HUMAN	2.27	1.61	-0.45	EDA_BOVIN;EDA_MOUSE
		TCR-α	TCA_HUMAN	2.29	1.85	-0.46	TCA_MOUSE
		Ig-α	CD79A_HUMAN	2.29	2.15	-0.56	CD79A_CANFA;CD79A_BOVIN; CD79A_MOUSE
		TREM-1	TREM1_HUMAN	2.18	1.00	-0.73	TREM1_PONAB;TREM1_PIG; TREM1_BOVIN;TREM1_MOUSE
		caveolin-3	CAV3_HUMAN	2.26	3.86	-1.72	CAV3_BOVIN;CAV3_PIG; CAV3_RAT;CAV3_MOUSE
		CD3-γ	CD3G_HUMAN	2.09	2.62	-1.89	CD3G_MACFA;CD3G_PIG; CD3G_RAT;CD3G_MOUSE; CD3G_BOVIN;CD3G_SHEEP
		CD3-ζ	CD3Z_HUMAN	2.04	2.10	-2.04	CD3Z_PIG;CD3Z_SHEEP; CD3Z_RABIT;CD3Z_MOUSE
		Fc-γ	FCERG_HUMAN	2.03	3.09	-2.60	FCERG_MACFA;FCERG_RAT; FCERG_MOUSE;FCERG_PIG; FCERG_CAVPO;FCERG_BOVIN
		CD3-ε	CD3E_HUMAN	1.90	1.95	-3.38	CD3E_MACFA;CD3E_CANFA; CD3E_RABIT;CD3E_BOVIN; CD3E_PIG;CD3E_FELCA; CD3E_MOUSE;CD3E_SHEEP; CD3E_CHICK
		mlgM	MUCM_RABIT	2.05	4.30	-3.40	MUCM_MOUSE;MUCM_ICTPU
		CD3-δ	CD3D_HUMAN	1.89	1.61	-3.43	CD3D_MACFA;CD3D_PIG; CD3D_RAT;CD3D_MOUSE; CD3D_SHEEP;CD3D_BOVIN
		IREM-2	CLM2_HUMAN	2.04	4.37	-3.56	CLM2_MOUSE
		G-CSFR	CSF3R_HUMAN	1.90	2.60	-3.73	CSF3R_MOUSE
		DAP12	TYOBP_HUMAN	1.79	2.02	-4.93	TYOBP_MACMU;TYOBP_PANTR; TYOBP_BOVIN;TYOBP_MOUSE; TYOBP_RAT;TYOBP_PIG
		NKG2C	NKG2E_HUMAN	1.95	5.50	-5.62	NKG2E_PANTR
		FXYD2	ATNG_HUMAN	1.72	2.94	-6.40	ATNG_RAT;ATNG_MOUSE; ATNG_SHEEP;ATNG_BOVIN; ATNG_XENLA
		CD200RLa	MO2R2_HUMAN	1.68	4.48	-8.39	MO2R2_MOUSE
		GPIX	GPIX_HUMAN	1.53	0.10	-9.18	GPIX_MOUSE

B	Cases of 8 sole hits between full length and TM-only sequence	TREM-2^a^	TREM2_HUMAN	1.91	2.47	-3.52	TREM2_MOUSE
		mlgA	IGHG3_MOUSE	2.04	4.45	-3.65	IGHG3_HUMAN
		Oscar	OSCAR_MOUSE	1.88	2.66	-3.95	OSCAR_HUMAN
		MPL	TPOR_HUMAN	1.80	2.28	-4.86	TPOR_MOUSE
		TREM-2^b^	TREM2_HUMAN	1.87	4.18	-5.25	TREM2_MOUSE
		GPVI	GPVI_HUMAN	1.57	3.84	-9.75	GPVI_MOUSE
		SILR-β	PILRB_HUMAN	1.57	4.16	-10.13	PILRB_MOUSE
		Ig-β	CD79B_HUMAN	1.29	11.26	-28.63	CD79B_MOUSE

There are in total 27 valid cases of functionally-related single-spanning membrane proteins from [34], out of which 19 cases are complete matches (section A) and 8 cases are sole hits (section B). The gene name and the UniProt ID of the seed sequences are given in column 3 and 4 respectively. The sequence complexity *x*_*c*_, hydrophobicity *x*_Φ _and z-score of the seed sequences are given in column 5, 6 and 7 respectively. The last column gives the orthologues of the seed sequences. For the 19 cases of complete matches, the TM-only sequences are able to retrieve their orthologous helices where their z-scores range from -9.18 to -0.10. For the other 8 cases of sole hits, they have failed to retrieve their orthologues. Their z-scores are between -28.63 to -3.52. The conflict zone between the 19 exact matches and 8 sole hits spans from -9.18 to -3.52. Note that TREM-2^a ^(Table one of [34]) and TREM-2^b ^(Table one of [34]) denote the same full length sequence but with a slight variation in the borders of the TM segment. Regardless of the variant, the conclusions remain unchanged.

Out of the 19 exact matches, 9 have z-scores above the twilight zone and hence they contain complex TM helices. These exemplary cases show that complex helices do exist in single-spanning membrane proteins. Most importantly, they can confer homology information instead of just serving as anchors (due to physiological requirement), thereby justifying the extension of the sequence homology concept for the single-spanning cases. For the 8 cases of sole hits, 3 of them have simple helices since their z-scores fell below the twilight zone. Fundamentally, these 3 cases demonstrate the lack of ancestry information in simple TM helices.

### Multi-spanning membrane proteins can harbor simple TM helices

The TCDB (Transporter classification database) [14,15] contains 656 distinct families but only 326 families that have TM annotations are useful for our further investigation. The total number of their TCDB entries is 2202. For each entry, the original sequence is first searched against the SwissProt database (dated 26-12-2010) with BLASTP [9]. Next, two more searches are performed; one for a masked version where the simple TMs are masked below a z-score threshold and another for a control version where the complex TMs are masked above the same threshold. The masking replaces the TM sequence positions with a continuum of 'X's. The z-score thresholds are set at various f = 0.84, 1.0, 1.282, 1.645. 1.98 (equivalent to false-negative rates of 20%, 16%, 10%, 5% and 2.5% taken with respect to the UniProt-derived functional TM-helices), where the larger false-negative rates indicate more aggressive z-score thresholds (more masking).

In the search results of the original sequence, hits with E-values more than 0.001 are discarded. In addition, hits without TCDB classifications are also removed. For the remaining hits, their corresponding alignment scores from the masked and control sequences are retrieved from the other two search results. Thus, each hit should be associated with a TCDB classification and three alignment scores from the original, masked and control sequences. The respective raw results are available as additional files.

This information is used to compute the number of true-positive, false-negative and false-positive hits. Since alignment scores contributed by simple TMs can be considered more dubious, the smallest alignment score representative of a true hit is determined from the minimum of the masked scores that have the same classification as the seed TCDB entry of the search. This minimum score is the cutoff used for the computation of false-discovery rate and sensitivity of the search for each TCDB entry (see methods). Given that a hit has the same classification as the TCDB entry, it is considered a true-positive if its score is equal or above the cutoff, otherwise it is a false-negative. Meanwhile, if a hit does not share the same classification as the seed entry, it is a false-positive if its score is equal or above the cutoff, otherwise it is a true-negative. Note that the sensitivity of the search for the masked sequence is always equal to 1.00 since the cutoff was derived based on its search results.

**Figure 6:**
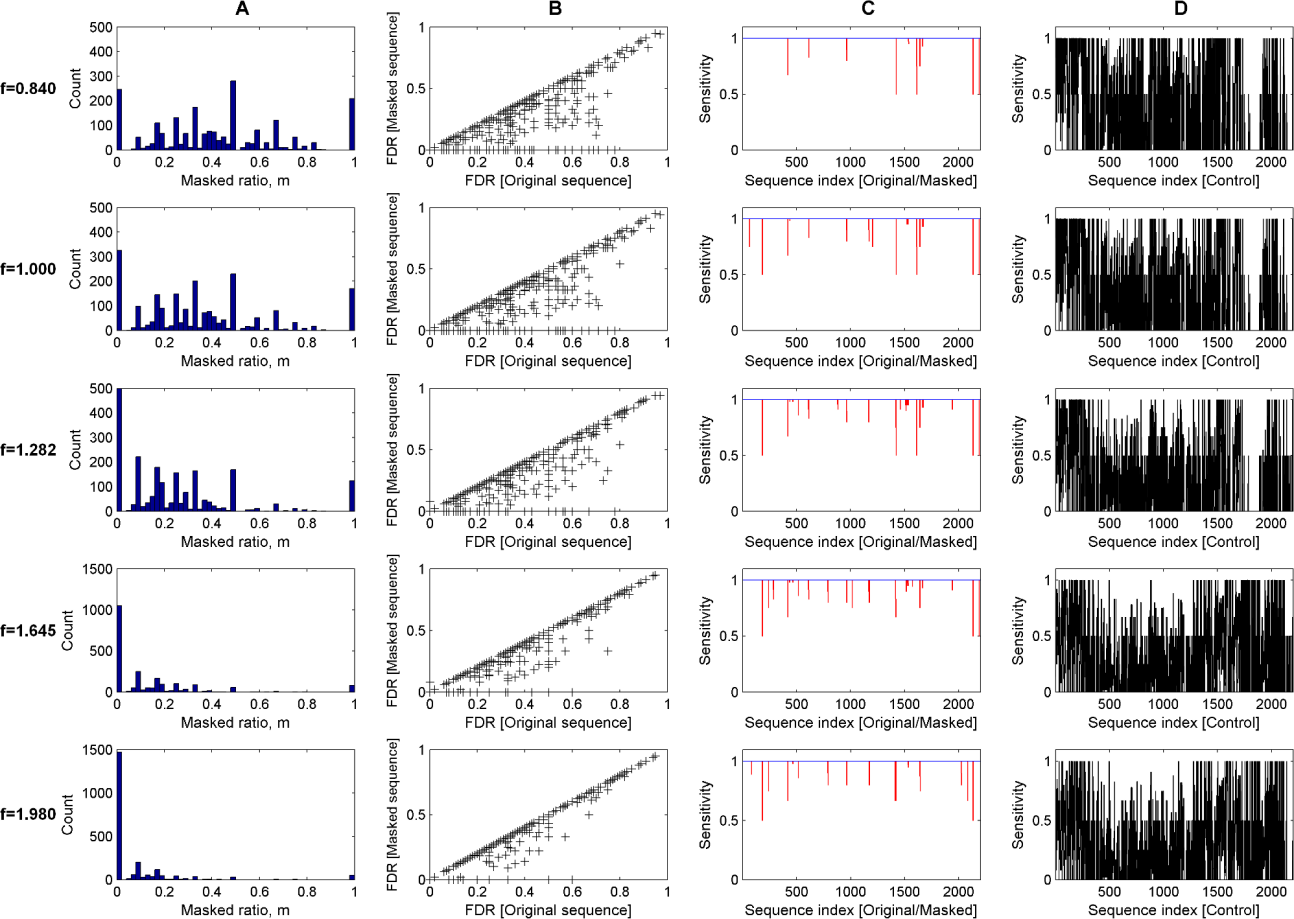
**Effects on the homology searches for TCDB with the removal of simple and complex TM helices**. The performance of the homology searches for 2202 TCDB entries between their original and masked sequences are shown for the z-score thresholds of f = 0.840, 1.000, 1.282, 1.645 and 1.980 respectively. The corresponding false-negative rates are 20%, 16%, 10%, 5% and 2.5% respectively. Figure 6A shows the masked ratios (m = number of masked TMs over total TMs) of the 2202 TCDB entries. The median mask ratios of the TCDB entries are 0.41, 0.33, 0.18, 0.08 and 0.00 for f = 0.840, f = 1.000, f = 1.282, f = 1.645 and f = 1.980 respectively. The non-zero mask ratio means that some TM helices in the multi-spanning entries are considered simple. The corresponding total number of entries (where 0 < m < 1) are 1747, 1705, 1533, 1071 and 680 respectively. On average, each masked TCDB entry has about 9 to 10 TM helices. Therefore, most TCDB entries are multi-spanning. Figure 6B shows that the false-discovery rates of the searches for the masked sequences are at least equal or less than that of their corresponding full sequences at a comparable sensitivity. This means that the masking of simple TMs can improve the false-discovery rates of the searches. This trend is independent of the different z-score thresholds that influence the level of masking (most masking at false-negative rate of 20% and least masking at false-negative rate of 2.5%). Figures 6C and 6D show the sensitivity plots of the searches for the 2202 TCDB sequences. The red, blue and black lines represent the sensitivities of the original, masked and control sequences respectively. The sensitivity of the original (red) and masked (blue) sequences are comparable at 1.0 for most of the sequences. At a sensitivity of 1, the number of false-negatives is zero. On the other hand, the sensitivity of the search for the control sequences (where the complex TMs are masked) deviates greatly from the sensitivity of 1. This implies that the masking of complex TMs has a detrimental effect on the TCDB classification.

Figure 6A shows a series of histograms of the mask ratios (m = number of masked TMs over total TMs per TCDB entry) for the z-score threshold settings of f = 0.840, 1.000, 1.282, 1.645 and 1.980. The corresponding total number of entries where TMs are partly masked (i.e., where 0 < m < 1) are 1747, 1705, 1533, 1071 and 680 respectively. The corresponding median mask ratios of the respective TCDB entries are 0.41, 0.33, 0.18, 0.08 and 0.00 depending on z-score threshold. The overwhelming majority of TCDB entries are multi-membrane-spanning with an average of 8 TM helices per entry. The non-zero mask ratios imply that some TM helices in the multi-spanning entries can be considered simple.

Table 4 shows the extremities of the effects of masking where mask ratio of 0 denotes that a TCDB entry has none of its TM helices masked (i.e. all TM helices are complex) and mask ratio of 1 denotes that all TM helices are masked (i.e. all TM helices are simple). The average number of TM helices in fully-masked entries ranges from 1.1 to 1.8 while the average number in fully-unmasked entries is between 4.4 and 7.3. On average, the masking of TM helices is more aggressive with entries having a low number of TM helices. In other words, simple TM helices are more dominant in single-spanning membrane proteins than the multi-spanning ones.

**Table 4:** Number of fully masked/unmasked TCDB entries and the corresponding average number of TM helices at various z-score thresholds of f = 0.840, 1.000, 1.282, 1.645 and 1.980

Mask ratio, m	f = 0.840		f = 1.000		f = 1.282		f = 1.645		f = 1.980	
	
	No. of entries	Avg no. of TMs	No. of entries	Avg no. of TMs	No. of entries	Avg no. of TMs	No. of entries	Avg no. of TMs	No. of entries	Avg no. of TMs
0.0	246	4.4	326	4.8	546	5.8	1050	6.9	1470	7.3
1.0	208	1.8	170	1.5	123	1.2	79	1.1	49	1.1

The two mask ratios of m = 0 and m = 1 correspond to the extreme situation where the TM segments in a TCDB entry is fully unmasked or fully masked respectively (column 1). The column pairs (2,3), (4,5), (6,7), (8,9) and (10,11) denotes the number of TCDB entries and the average number of TM helices (total number of TM helices in the entries/total number of entries) given the associated mask ratio and the z-score thresholds of f = 0.840, 1.000, 1.282, 1.645 and 1.980 (corresponding to false-negative rates of 20%, 16%, 10%, 5% and 2.5%). The average number of masked helices ranges from 1.1 to 1.8 while the average number of unmasked helices is between 4.4 and 7.3. This means that the fully masked TCDB entries are almost exclusively the single-spanning ones while the fully unmasked entries are often the multi-spanning ones.

Figure 6B shows a series of plots of the false-discovery rates between the masked and original sequences of the 2202 TCDB entries as a function of z-score threshold. Regardless of the threshold values, the false-discovery rates of masked sequences for some of the TCDB entries are less than or equal to that of their corresponding original sequences. Thus, it is positive for the similarity search to exclude simple TMs since more false-positive hits are suppressed. For more aggressive z-score thresholds (smaller f), more TCDB entries undergo extensive TM masking and, subsequently, experience a decrease in/an improvement of their false-discovery rates.

The improvement of false-discovery rates that is accompanied by the removal of simple TMs is not compromised by the sensitivity of the search. Sensitivity is best described as the power of a statistic test which is its ability to reject the null hypothesis when it is false. Maximum sensitivity is numerically defined as 1. Figure 6C show the sensitivity of the 2202 entries for the z-score thresholds of f = 0.840, 1.000, 1.282, 1.645 and 1.980. The red and blue lines represent the sensitivities of the original and masked sequence searches. The sensitivities of both searches hardly vary for most of the entries. In those occasional cases where they do, the sensitivities of the original sequences are less than that of the masked ones. On the other hand, the sensitivities (see Figure 6D) of the control sequences (i.e., where the complex TMs with z-score above the threshold are masked) fluctuate wildly across the sensitivity axis. This shows that the masking of complex TMs decreases the sensitivity of the search. In hindsight, this investigation shows the importance of complex TMs as homology pieces and the redundancy of the simple TMs for homology searches.

### Frequency of simple helices decreases as number of spanning TM helices increases

Although simple TM helices can be expected in either single-spanning or multi-spanning membrane proteins, their rate of occurrence remains unclear. Here, we attempt to compute the true-negative rate and the estimated number of the simple TM helices when given any multi-spanning proteins with a fixed number of TM helices.

**Figure 7:**
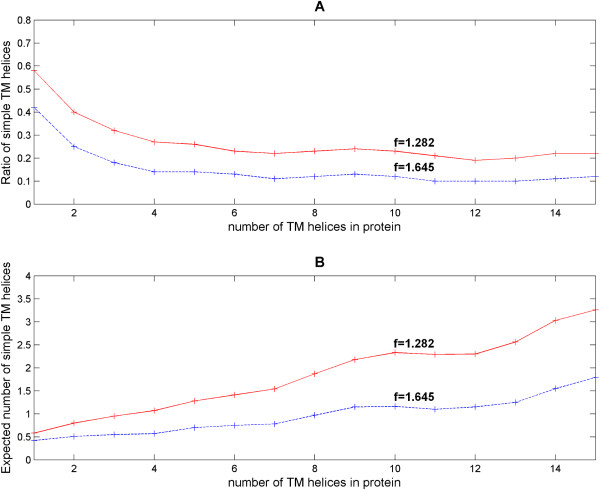
**Rate of occurrences and expected number of simple TM helices with increasing number of TM helices in membrane proteins**. Figure 7A shows the general trend that the rate of occurrences (in terms of ratio of simple TM helices) decreases exponentially as the number of TM helices increases in the protein. The ratios stabilize beyond 6 TM helices. Note that the trend is independent of the z-score thresholds of f = 1.282 (false-negative rate of 10%; see red lines) and f = 1.645 (false-negative rate of 5%; see blue lines). On the other hand, Figure 7B shows that the expected total numbers of simple helices do not plateau with increasing TM helices. Generally, the number of expected simple helices *sh *is about proportional to the number of TM helices *nTM *in the protein. The relationship between these two values is *sh *= 0.218*nTM *(goodness of fit is *R*^2 ^= 0.93) for f = 1.282 and *sh *= 0.112*nTM *(goodness of fit is *R*^2 ^= 0.82) for f = 1.645.

Hence, the UniProt sequences and their associated annotations (based on UniProt annotation file dated 16-09-2010) were retrieved based simply on the keyword FT_TRANSMEM. Fifteen datasets were created from 1 to 15 TM segments (the number of available proteins with more TMs is too small for this assessment). For each protein, the z-score of all TMs is calculated using the parameterization of the UniProt-derived functional TM-helices datasets. A TM with a score below threshold is considered simple. In Table 5, we list the fractions of the number of complex and simple TMs relative to the total number of TMs found in the set (depending on z-score thresholds with f = 1.282 (false-negative rate of 10%) and f = 1.645 (false-negative rate of 5%)). The latter ratio (Figure 7A) can be used to compute the average expected number of simple TMs in a protein with a given number of TM regions (Figure 7B).

**Table 5:** Rate of occurrences/expected number of simple TM helices in membrane proteins with fixed number of TM helices

No. of TMs	f = 1.282			f = 1.645		
	
	Ratio of complex TMs	Ratio of simple TMs	Expected no. of simple TMs	Ratio of complex TMs	Ratio of simple TMs	Expected no. of simple TMs
1	0.42	0.58	0.58	0.58	0.42	0.42
2	0.60	0.40	0.80	0.75	0.25	0.51
3	0.68	0.32	0.95	0.82	0.18	0.55
4	0.73	0.27	1.07	0.86	0.14	0.57
5	0.74	0.26	1.28	0.86	0.14	0.70
6	0.77	0.23	1.41	0.87	0.13	0.75
7	0.78	0.22	1.54	0.89	0.11	0.78
8	0.77	0.23	1.87	0.88	0.12	0.97
9	0.76	0.24	2.18	0.87	0.13	1.15
10	0.77	0.23	2.33	0.88	0.12	1.16
11	0.79	0.21	2.29	0.90	0.10	1.10
12	0.81	0.19	2.30	0.90	0.10	1.15
13	0.80	0.20	2.56	0.90	0.10	1.25
14	0.78	0.22	3.03	0.89	0.11	1.55
15	0.78	0.22	3.26	0.88	0.12	1.79

The rate of occurrences and expected number of simple TM helices are computed for 15 sets of UniProt-derived membrane entries grouped by the number of TM helices they contain. The number of TM helices for each set is given in column 1. Column pairs (2,3) and (5,6) gives the ratios of complex and simple TM helices found in each set based on the z-score thresholds of f = 1.282 (false-negative rate of 10%) and f = 1.645 (false-negative rate of 5%) respectively. Similarly, column 4 and 7 gives the expected number (No. of TMs × ratio of simple TMs) of simple TM helices in each set at those z-score thresholds.

The general trend is that the ratios of simple TM helices occurrence decrease exponentially as the number of TM helices increases in the protein. Beyond 6 TM helices, the frequency of occurrence almost stabilizes (Figure 7A). The trend is independent of the various z-score thresholds. Unlike the expected frequency of simple helices, the expected total numbers of simple helices do not plateau (Figure 7B). This is because the number of expected simple helices is about proportional to the number of TM helices in the protein.

### Examples of simple and complex TM helices in known membrane proteins

For the introductory example of 7-TM rhodopsin protein (P02699), the z-scores of its seven TM helices are -3.23 (TM-1), 0.05 (TM-2), 0.33 (TM-3), 0.43 (TM-4), -9.00 (TM-5), -2.67 (TM-6) and 2.57 (TM-7) respectively. Meanwhile, the z-score twilight zone is between -5.41 (f = 1.645) and -3.29 (f = 1.282). Hence, z-scores below -5.41 are considered simple while z-score above -3.29 are complex. Consequently, only TM-5 of rhodopsin is considered simple. This collaborates well with the expected number of simple TM of between 0.78 and 1.54 for membrane proteins with 7 TM helices (see Table 5). So far, the functional role of this TM segment has not been well established whereas the Gly51 in TM-1 and Gly89 in TM-2 have been linked to the retinal degenerative disease autosomal dominant retinitis pigmentosa [29] while Glu113 in TM-3, Ala169 in TM-4, Trp265 in TM-6 and Lys296 in TM7 are functionally important [28,30].

Next, the *Escherichia coli *GlpG (P09391), a 6-TM bacterial rhomboid protease, has an active cleft that is characterized by the catalytic residues His150 in TM-2, Ser201 in TM-4 and His254 in TM-6. Furthermore, it is suggested that both TM-1 and TM-3 provide only a structural role while TM-5 possesses conformational flexibility given that the substrate enters through the gap between TM-2 and TM-5 [22-24]. Correspondingly, the z-scores of the TM helices are -3.59 (TM-1), -0.72 (TM-2), -2.52 (TM-3), 1.18 (TM-4), -0.95 (TM-5) and 3.27 (TM-6) respectively. All helices except TM-1 are considered complex for a z-score threshold of above -3.29 (f = 1.282). Interestingly, within the complex helices, the higher z-scores are able to differentiate the functionally-related helices (TM-2, TM-4, TM-6) from the structurally-related ones (TM-3, TM-5).

Here, an example of the 'in-betweener' protein *Escherichia coli *aspartate receptor (Tar; P07010) is a chemotaxis receptor in bacteria. It is composed of 2 TM helices (TM-1 and TM-2) and 3 bacterial domains as annotated by SMART [20,21] (SM00319-TarH: ligand binding domain, SM00304-HAMP: signal transduction domain, SM00283-MA: methyl-accepting chemotaxis-like domain). This receptor forms a homodimer through helix interactions between TM-1 of two monomers, without any involvement of TM-2 [41,53]. In fact, our z-score computations also show that TM-1 of Tar (z-score of -3.36) is indeed more important than its TM-2 (z-score of -24.48). Given the twilight zone of -5.41 (f = 1.645) and -3.29 (f = 1.282), TM-2 of Tar is considered simple. The expected number of simple TM helices is slightly underestimated at between 0.51 and 0.80 for membrane proteins with 2 TM helices (see Table 5).

Colicin is an interesting protein with two forms, one soluble form with known 3D structure (accession 1col [54]) where two nascent TMs (helices 8 and 9) are incorporated into the volume of a globular structure and another membrane-bound, pore-forming form [55]. Existing experimental evidence shows that helix 8 can interact with the colicin immunity protein of the host [56,57]. In agreement, we find that helix 8 is a complex TM (z-score of -0.82 for colicin Y/accession Q9KJ98 and similar for other colicins), whereas helix 9 looks more like a simple TM (z-score of -4.38).

Finally, the z-score of another introductory example the human APMAP (Q9HDC9) is found to be -2.84. Interestingly, its z-score is above the twilight range and hence its sole TM helix is considered complex. Interestingly, the TM-only sequence search of the human APMAP (as a seed orthologue against SwissProt dated 26-12-2010) returns 4 out of 6 of its orthologous APMAP (APMAP_RAT, APMAP_MOUSE, APMAP_BOVIN, APMAP_CHICK, APMAP_DANRE, APMAP_SALSA). This means that the TM helix is likely to contain ancestry information and hence functionally important. But so far, the functional aspect of the APMAP TM helix has not been characterized and presents an interesting case for further experimentation.

### Application of the proposed necessary z-score criterion for simple TM suppression in domain models highlighted in our previous work

In our previous work [1], we described 15 exemplary cases of protein domain models in Pfam that pick up significant false hits or missed true hits due to the inclusion of TM segments into the model. These domains listed in Table 6 were compiled from Supplementary Tables S1, S2 and S3 of [1]. Here, we wish to examine the relationship between the type of TM segments (whether simple or complex) embedded in these domain models and the associated false and missed hits through our z-score criterion. Clearly, none of the hits described in the Supplementary Tables S1, S2 and S3 from [1] would surface in a database search if the respective TM segments were excluded from the respective models.

**Table 6:** Problematic Pfam domains that picked up false-positive hits and missed false-negative hits in HMMER2 searches due to the inclusion of TM segments

Domain name	Validated TM helices, reference(s)	HMM TM sequence	*x*_*c*_	*x*_Φ_	z-score
PF01537.9 : Herpes_glycop_D (Herpesvirus glycoprotein D)	372-393, ref. [80]	VIIGIVVLALLIGAIIVGVVYY	1.20	4.72	-19.34

PF03381.7 : CDC50 (ligand-effect modulator 3/CDC50)	318-340, ref. [81]	PFLGIAYLVVGGLCLVLGIVFLI	1.66	2.78	-7.37

PF00690.18 : Cation_ATPase_N (Cation transporter/ATPase, N-terminus)	66-87, ref. [82]	DPLVLLLLAAAIISALDFVLGG	1.68	2.38	-6.83

PF00482.11 : GSPII_F (Bacterial type II secretion system protein F domain)	118-136, ref. [83]	LLLIVALLILLLLLAILLP	0.55	10.89	-53.23

PF01569.13 : PAP2 (type 2 phosphatidic acid phosphatase)	129-143, 156-172, ref. [84]	LLGLLLLLLALLVGLSRVY, LAGALLGALIAALVLLFVR	1.23, 1.44	4.48, 2.35	-18.43, -11.49

PF01001.11 : HCV_NS4b (Hepatitis C virus non-structural protein NS4b)	124-143, 156-179, ref. [85]	RVLVDVLGGYEAAVNAASLT, DLVNLLPALLSPGASVVGVALALI	2.50, 2.14	4.98, 1.45	***3.50***, ***-1.08***

PF08510.4 : PIG-P (phosphatidylinositol N-acetylGlucosaminyl transferase subunit P)	8-24, 44-67, ref. [86]	GFVLYILSQLAFILYLLWAF, YWALAIPIYLLVALIFGYVVYFLY	2.19, 1.85	5.77, 5.35	-3.99, -6.72

PF01105.15 : EMP24_GP25L (Endoplasmic recticulum and golgi apparatus trafficking proteins)	142-162, ref. [87]	WWSIIQLLVLVGVSVFQVYYL	1.71	4.83	-8.09

PF04387.6 : PTPLA (protein tyrosine phosphatase-like protein)	89-106, 138-155, refs. [88,89]	YTLFIVLYPLGVTSELLTVY, LIIALMLIYIPGFYQLYSH	2.34, 2.47	2.87, 3.28	***-0.79***, ***-0.99***

PF01299.9 : Lamp (Lysosome-associated membrane glycoprotein)	304-327, ref. [90]	LIPIAVGAALAGLVLIVLIAYLIG	1.44	3.50	-12.34

PF02416.8 : MttA_Hcf106 (sec-independent translocation mechanism protein)	1-19, refs. [91,92]	IGIPELLIILVVALLLFGP	1.20	5.42	-20.07

PF00672.17 : HAMP (cytoplasmic helical linker domain)	1-15, ref. [93]	LLLVLLIALLLALLLALLL	0.73	10.01	-43.85

PF01544.10 : CorA (CorA-like Mg2+ transporter protein)	342-362, 377-401, ref. [94]	LLTVGTTIFAPLTLIAGIYGM, YGYPLVLGLMAILAIVLFLIILSYF	2.36, 1.65	1.70, 7.03	***-0.25***, -11.97

PF00558.11 : Vpu (Vpu protein)	6-28, ref. [95]	IIGLIALIVALIILAIVVWTIVI	1.04	8.09	-28.92

PF04901.5 : RAMP (Receptor activity modifying family)	85-106, ref. [96]	VLLPLIVVPITLTLLLTALVVW	1.31	8.50	-21.96

The first column lists the fifteen problematic Pfam domains from our previous work [1] where they picked up significant false hits or missed true hits due to the inclusion of TM segments in the model. These domains were compiled from supplementary Tables S1, S2 and S3 of [1]. The second column gives the regions of the validated TM in the Pfam HMM (hidden markov models) models with respect to the given references. In a nutshell, the HMM sequences were aligned to the sequences provided in the literatures for the demarcation of the TM segments in the HMM models. The third column denotes the emitted HMM sequence of the TM segments. The last three columns give the complexity *x*_*c*_, hydrophobicity *x*_Φ _measures and subsequent z-scores of the TM segments. Note that the complex TM segments are highlighted in bold italic.

Out of the 15 problematic domain model, 10 are single-TM-spanning and the other 5 are two-TM-spanning. Given a twilight zone of -5.41 (f = 1.645) to -3.29 (f = 1.282), a TM helix with a z-score of above -3.29 is considered complex while below -5.41 is considered simple. Hence, all the single-spanning domain models have simple TM helices since their z-scores are between -53.23 and -6.83. For 3 of the two-TM-spanning models, i.e., the PAP2 (PF01569.13), the PIG_P (PF08510.4) and the CorA (PF01544.10) domain models, each model has at least one simple TM segment. This was the expected rate of occurrence based on Table 5. Among the TM segments of these 3 models, 4 are considered simple, 1 is within the twilight zone and 1 is complex. The particular complex TM segment from CorA does not form any alignment with respect to its hit (AAO72700.1). Therefore, for all the above mentioned models, the associated false and missed hits were solely attributed to the simple TM segments.

For the remaining two-TM-spanning models, PTPLA (PF04387.6) and HCV_NS4b (PF01001.11), the z-scores of their TM segments indicate that they are complex. To recapitulate, the fragmentary-mode hit (XP_001939830.1) that was detected by HCV_NS4b only has a partial alignment to the second TM segment of the model ('LLSPGASVVGVALALI'). Similarly, the global-mode hit (EAY72555.1) only has an alignment to the first TM segment of the domain model PTPLA. In both cases, the alignment to the other complex TM segment were absent, hence these hits remained as false-positive cases.

In hindsight, our z-score works as a necessary criterion for the exclusion of simple TM segments from domain models with the desirable consequence of suppressing many false-positive hits in similarity searches. At the same, the criterion is not sufficient to suppress all false-positive hits that might originate from chance alignments of TM regions with hydrophobic stretches.

## Discussion

### The notion of simple and complex TM helices

At the beginning of this work, there was the observation that TM regions are heterogeneous in the hydrophobicity/sequence complexity space. We found evidence that there are subpopulations of TM segments. For one of them, the TMs are more hydrophobic than average TMs and they have a lower complex sequence enriched in aliphatic hydrophobic amino acids. Correlating this to an experimentally-derived hydrophobicity scale [32,33,35], these TM helices have a higher propensity for membrane insertion than the average ones. Originally, we tied these TM regions to single TM proteins. As a trend, they are indeed more frequently occurring in proteins with just one or very few TMs; yet, we found that they may also be present in multi-spanning membrane proteins. Since these TM regions are actually mere hydrophobic anchors, we call them simple TMs.

On the other end, there are TM regions that are enriched in charged (RDEH), structural (GP) and aromatic (FW) residues relative to the average TM. Thus, these TM regions are not so hydrophobic and they have higher sequence complexity. The listed residues are expected to have some role in the biological function of the TM proteins, for example, enabling the TM region to participate in ligand binding, active sites, signal transduction, structural packing of TM helices and complex assembly. We call these functional/structural TMs complex. As a trend, complex TMs are more frequent in multi-TM proteins but they may also occur in single-membrane spanning proteins.

It should be mentioned that the observation of TMs with differing hydrophobicity and some of the structural and functional implications have already been widely discussed in the literature. For example, amphipatic helices in multi-spanning TM proteins are a subtype of complex TMs. Their hydrophobic moments influence membrane protein folding [58] and their sequence patterns can be used to predict the burial status of residues in the TM proteins [59]. We wish to emphasize that, in this work, we derived the notions of simple and complex helices from an unbiased analysis of the distribution of annotated TM helix regions in the hydrophobicity/sequence complexity space and its differences depending on the number of TM regions per protein. Thus, we have a statistical basis for 'simple' and 'complex' TM helix distinction and, thereby, a justification for their separation.

In this work, we propose a z-score framework (based on hydrophobicity and sequence complexity assessment) for this purpose (see equations (1) and (3)). Since the distribution of simple and complex TM helices in this space is overlapping, it is only possible to derive a necessary criterion for the simple helices (z-scores below a low threshold) and a sufficient criterion for the complex helices (z-scores above a high threshold). Clearly, there is a twilight zone for TM helices with intermediate z-scores which are difficult to classify without *a priori *knowledge of the protein's evolution and function.

For practical purposes, we suggest to define a twilight zone of z-score between -5.41 to -3.29 (computed at 5% (f = 1.645) and 10% (f = 1.282) false-negative rates with respect to the UniProt-derived functional TM helices). TM-helices with z-scores above -3.29 are flagged as complex helices while those below -5.41 as simple.

### Simple and complex TM helices occur in either single- or multi-spanning membrane proteins at different frequencies

We have shown the possibility of complex helices in single-spanning membrane proteins. Instead of being merely anchors, the sequences of the complex helices of the disease-associated proteins and immune receptors were able to confer sufficiently high homology information. On the other hand, the occurrence of simple helices in the multi-spanning membrane proteins has been illustrated via the TCDB database where we showed that the masking of simple helices in homology searches can improve the false-discovery rate of the classification without compromising on the sensitivity of the searches. Yet, the masking of the complex ones wreaked havoc to the sensitivity.

Taken together, the existence of either simple or complex TM helices in integral membrane proteins is independent of their topology. As a trend, we find that the frequency of occurrence of simple helices in the membrane protein is proportional to the number of TM helices found within. To emphasize further, the TM helix of a single-spanning membrane protein is expected to be simple more than ~60% of all cases. For a multi-spanning protein, the frequency of occurrence of simple helices decreases as its total number of helices increases. This frequency stabilizes to around 0.3 (for a false-negative rate of 10%) when the total number of spanning helices reaches to 6 and beyond.

### About the extension of the sequence homology concept to membrane proteins

Decades before the genomic era, the principle of inferring evolutionary history from sets of homologous protein sequences (e.g. 1964, fibrinopeptides [60]; 1967, cytochrome c [61]) to build believable phylogenetic trees has already been established [62,63]. In the same period, another principle for inferring homology through the trinity of sequence-structure-function has also been successfully applied to unknown sequences with high sequence similarity to characterized structures (e.g. 1967, lactalbumin model is built using the X-ray coordinates of lyzosome where the two sequences are concluded to be homologous for being 35% identical [64]; 1986, angiogenin is homologous to pancreatic ribonuclease where the X-ray structure of the latter is known [65,66]). Essentially, these principles that govern the modus operandi of the present day sequence homology concept remain unchanged.

Though homology has the precise meaning of "having a common evolutionary origin" [67], it also carries the loose meaning of "possessing sequence similarity or being matched" when translated into computerized homology searches. Maybe, it would be more appropriate to use the term "sequelog" coined by Varshavsky [68] in this context. In reality, homology between sequences is always a hypothesis while similarity, being a measurable fact, can be rationalized either as chance, convergent evolution or common ancestry [69-71]. While similarity by chance can be eliminated via stringent statistical criterion (e.g. E-value cutoff), ambiguity between convergent evolution and common ancestry, both inferred from similarity, can arise and this requires extra considerations. As a guide, we must be mindful in distinguishing between long stretches of similarity (typical for the construction of phylogenies) from those local resemblances that are physiologically constrained for particular side chains to form certain rudimentary structures (e.g. membrane spanning stretches from non-polar residues; turns and loops from polar ones) [72].

Fast forward into the present genomic era, alignment tools (e.g. BLAST [9], HMMER [73,74]) and domain libraries (e.g. SMART [20,21], Pfam [12,13]) have become the de-facto components of many automated annotation pipelines to detect the homology and hence, to infer the functions of many unknown sequences accumulating in the relentlessly growing sequence databases. The collateral damage from such convenience is an overestimation of homology since statistically significant similarities are interpreted as homology without an alternate exit as convergence.

Although there have been previous attempts to create separate BLOSUM-like matrices, like PHAT [75] and SLIM [76], to perform homology searches for membrane proteins, their performance is limited to clearly defined TM protein families preferably with structural information. These non-symmetric matrices are generally not applicable on uncharacterized sequences when the choice of the score matrix to use for which residues is unclear given unknown structural domains of the sequences. In a nutshell, a generalized methodology to perform homology searches for membrane proteins that also naturally extends the present sequence homology concept does not exist. Despite that, the sequence homology concept has been silently extended to membrane proteins, most commonly through an automated annotation pipeline. As a result, the unjustified sequence similarity of simple TM helices to unrelated sequences can transform itself into an eventual annotation error propagation disaster [77,78]. In addition, the exaggerated E-values of HMMER2 [73,74] embedded in annotation pipelines might further complicate the issue [79].

To recapitulate, the question of whether TM helices originate from convergent evolution or common ancestry will remain a topic of theoretical debate. Instead, we explored the legal separation of simple (implying convergent evolution) and complex (implying common ancestry) TM helices in this work. We provide quantitative evidence that confirms their separability through the sequence complexity/hydrophobicity plot. The overlap between the functional TM-helices and the functional α-helices of globular domains (as a close structural analogue to the TM helix) in this plot serves as a first approximation for the applications of the sequence homology concept for the membrane proteins. Subsequently, extensive investigations with the single- and multi-spanning membrane protein sets (disease-related/immune receptors and TCDB) have reinforced that the sequence homology concept can indeed be generalized to the complex TM helices regardless of the topology of the membrane protein. The practical key to the extension of the sequence homology concept to membrane proteins is the z-score criterion. The suppression of TM segments with low z-scores in similarity searches keeps the simple TM helices at bay since the latter (implying the results of convergent evolution) artificially inflate similarity scores that do not help in explaining homology.

### About the treatment of transmembrane helical segments in protein domain models

Homology has the elusive meaning of "possessing sequence similarity" when implemented as computerized homology searches. For these searches, statistically significant similarities can be misinterpreted as homology given the lack of consideration for convergence as an alternate explanation. For the membrane proteins especially, the ambiguity between common ancestry and convergent evolution is made worse because TM helices are, as a trend, homogenously hydrophobic.

Continuing previous work [1], we conclude that a group of TM segments (in the form of membrane anchors) from domain models most likely exhibits sequence similarity as a result of convergent evolution. We find that TM anchors and functional TM helices are indeed distinct in amino acid composition and, hence, different in sequence complexity and hydrophobicity. Essentially, this basis establishes the notion of simple (implying convergent evolution) and complex (implying common ancestry) TM helices in nature and, hence, the possibility of their separation with computational criteria.

Further investigations showed that the segregation of simple TM helices from the protein sequences has a positive impact on the sensitivity of homology searches. Most interestingly, complex TM helices can occur in single-spanning membrane proteins contrary to their role as mere anchors, though at a much lower frequency than the multi-spanning ones. In hindsight, complex TM helices can occur in membrane proteins regardless of their topology.

Finally, the existing sequence homology concept and computational framework can be extended to the membrane proteins when simple TMs are suppressed. A necessary criterion in the form of a z-score function was introduced for the purpose of identifying the simple TM helices (as well as the complex TM helices) within the sequences so that they can be properly identified prior to homology searches with methods based on sequence similarity. This is similar to applying sequence complexity filters such as SEG [10] in similarity searches. It should noted that, similarly to SEG that does not recognize all low-complexity sequence regions, our z-score criterion will detect many but not all TMs that might confuse searches for homologous sequences. In the end, a pure statistical consideration of computed homology relationships without looking at biological criteria [2] remains insufficient.

Pertaining to the comment of the reviewer of our previous article [1] (see Introduction), it is indeed not necessary to suppress complex TM segments within domain models collected in protein domain databases such as Pfam or SMART. Conflicts arise when simple TM regions in domain models support seemingly significant, yet false-positive alignments with their hydrophobic runs. The conceptual advance described here provides a z-score criterion to decide whether to include certain TM helices into models of protein domains.

## Conclusions

The trinity of similar sequence resulting in similarity of structure and function is the cornerstone of the sequence homology concept and the basis for functional predictions for uncharacterized sequences. However, it is not widely anticipated in the community that this principle is generally applicable only for protein sequence segments that represent globular structures but not for non-globular segments that frequently occur especially in proteins of eukaryotic origin. To apply sequence homology considerations on proteins with transmembrane regions (TMs), the original concept requires modification to prevent false homology inferences among sequences that have nothing in common except for extended stretches of hydrophobic amino acid residues. In this work, we classify TMs into simple and complex TMs. Whereas the former are essentially pure hydrophobic stretches that merely anchor the otherwise soluble protein to the membrane, the latter ones have some functional residues at the generally hydrophobic sequence background that give these TMs additional structural (intra-membrane complex-forming) or functional (ligand-binding, intra-membrane catalytic, etc.) role. We provide a quantitative criterion based on hydrophobicity and sequence complexity assessment to distinguish simple from complex TMs. We recommend masking simple TMs from query sequences prior to sequence similarity searches.

## Methods

### Derivation of sequence data sets

Six characteristics datasets were derived from SCOP alpha and membrane class (version 1.75) and UniProt annotation file (dated 16-09-2010). They are the functional TMs, membrane anchors, signal anchors, functional α-helices, signal peptides and low-complexity segments. Based on the UniProt annotations, all TM proteins (functional TMs, membrane anchors, signal anchors and multiple TMs) are extracted based on the feature table keyword FT_TRANSMEM. We found that the median length of all UniProt-derived TM helices is 21 residues. Globular sequences like functional α-helices and signal peptides are based on the FT_HELIX and FT_SIGNAL keywords respectively. Only RefSeq sequences were processed. In addition, substrings and repeated sequences were removed from each of the derived dataset via the cd-hit algorithm [39] (with options -n 5 -c 1).

#### Functional TM-helices

In our definition, a functional TM denotes a TM helix that contains residue(s) that is expected to confer the biological function of the protein analogous to globular proteins. Hence, these residues are expected to bind ligands, to be important for catalytic activity, etc. For the derivation of functional TMs, the TM entries are further mined for the following specific keywords FT_METAL, FT_BINDING, FT_ACTIVE. In the end, two sets of functional TMs were derived. One was from SCOP membrane class and another was from UniProt. The rationale is that the SCOP-membrane derived set would be more reliable since SCOP was manually curated though the sampling was expectedly limited (before and after the application of cd-hit, the number of sequences is 984 and 83 respectively). On the other hand, the UniProt-derived set, though much larger (Before and after the application of cd-hit, the number of sequences is 3923 and 1741 respectively.), was not expected to be free of annotation errors. Together, the two sets should give us an idea of the upper and lower bound for our computation results.

Generally speaking, it is thought that the TM helix in a single-spanning TM protein (e.g. for Type I and II membrane proteins) functions as a simple anchor (resulting from convergent evolution) and does not confer its biological function in contrast to its globular part in most cases. The signal and membrane anchor sets are representative of this class of TM helices. For the derivation of both sets, the protein sequences were first checked for a single annotation of FT_TRANSMEM to ensure these entries are single-spanning TM proteins.

#### Signal anchors

An additional description of 'Signal-anchor' was enforced. An anchor is considered at N-terminus if it is located on the first 100 amino acids position of its full length sequence. The first 100 positions should coincide with the positions of a signal peptide (expectedly at the N-terminus) if one occurs. Before and after the application of cd-hit, the number of sequences is 2280 and 1767 respectively. Out of the 1767 signal anchors, 372 signal anchors were predicted as signal peptides are predicted as signal peptides both by the Hidden Markov Model (HMM) and the neural network versions of SignalP. 1071 of the signal anchors are predicted by either version of SignalP. The remaining 324 examples are rejected as possible signal peptides by both versions. Out of them, 218 are N-terminal signal anchors (within the first 100 residues; see methods), 106 are more C-terminally located.

#### Membrane anchors

An additional description of 'Anchor' was enforced. Before and after the application of cd-hit, the number of sequences is 378 and 303 respectively. All except 15 of the resulting set of 303 membrane anchors are located on the C-terminal end of the proteins (i.e., beyond the first 100 residues). None of the remaining N-terminally located membrane anchors has a positive signal peptide prediction from either SignalP-HMM or SignalP-NN.

#### Functional α-helices

As close structural analogues to the α-helical functional TMs, a set of functional α-helices were derived from the entries under the SCOP alpha proteins class. Besides the FT_HELIX keyword, the SCOP entries were mined for the same keywords FT_METAL, FT_BINDING, FT_ACTIVE. Before and after the application of cd-hit, the number of sequences is 17193 and 1330 respectively.

#### Signal peptides

As close analogues to the signal anchor sets, a set of signal peptides were derived from the entries under the SCOP alpha and membrane proteins class. The SCOP sequence entries were checked against the UniProt annotation file for the FT_SIGNAL keywords. This resulted in 1664 sequences from SCOP alpha and membrane classes. After the application of cd-hit, only 262 signal peptides remained.

#### Low-complexity regions

The SEG algorithm with parameters (window size/trigger complexity/extension complexity) 25/3.0/3.3 is applied to the SwissProt sequence database (dated 26-12-2010) resulting in 577373 segments. After applying cd-hit for sequence redundancy removal and the subsequent suppression of sequence segments with less than 15 residues, 475207 low-complexity segments remained.

Whereas redundancy removal caused by substrings and repeated sequences is essential, introduction of a more stringent sequence identity threshold is not. Given the short length of about 20 amino acid residues per TM segment and the prevalence of aliphatic hydrophobic residues in them, very low sequence identity thresholds do not make sense since they would simply obscure datasets. Most importantly, the 85% threshold datasets do not give rise to any relevant change in the summary statistics compared with Table 1. For example, an 85% sequence identity threshold results in 1057 (UniProt-derived) and 79 (SCOP-derived) sequences respectively for the functional TM helices, 1427 for the signal anchors or 239 for membrane anchors. As an example for the row with sequence complexity window size 12 in Table 1 (section A), the results are 2.43, 0.30, 79, 2.39, 0.30 and 1057. For Table 1 (section B), we get 0.33, 2.95, 79, 0.46, 2.87 and 1057.

### The Shannon's entropy equation with IVL as a single group

Given that the amino acids I,V,L are considered as a single group while all others are considered as individual, the sequence complexity *x*_*c *_[10] is defined as

(5) $$x_{c}=-\overset{V}{\underset{i=1}{\sum }}\frac{m_{i}}{L}\left(\log_{2}\frac{m_{i}}{L}\right)$$

where *L *is the moving window size (i.e. size of 10, 12, 15, 18 in our work), *V *is the number of distinct groups of amino acids (i.e, 18 since IVL is considered as a group), *m*_*i *_is the number of amino acids that belongs to a particular group where *i *= 1..18.

### The regressed straight line and normal equation of the normalized sequence complexity and hydrophobicity datapoints

Given *n *data points (*x*_*c,i*_, *x*_Φ_,_*i*_) where *i *= 1...*n *and *x*_*c *_and *x*_Φ _are the sequence complexity and hydrophobicity measures respectively, one wishes to find the best straight line *x*_Φ _= *α *+ *βx*_*c *_that passes through these data points. The intercept *α *and slope *β *can be found by minimizing the sum of squared residuals given as $\overset{n}{\underset{i=1}{\sum }}\varepsilon_{i}^{2}=\overset{n}{\underset{i=1}{\sum }}{\left(x_{\Phi ,i}-\alpha -\beta x_{c,i}\right)}^{2}$. As a consequence, the estimates of the intercept *α *and slope *β *are given as

(6) $$\hat{\alpha }=\mu_{\Phi }-\hat{\beta }u_{c}\;\text{and}\;\hat{\beta }=\rho_{c,\Phi }\frac{\sigma_{\Phi }}{\sigma_{c}}$$

where (*u*_*c*_, *σ*_*c*_) and (*μ*_Φ_,*σ*_Φ_)are the mean/standard deviations of the sequence complexity and hydrophobicity respectively, and *ρ*_*c*, Φ _is the correlation between complexity and hydrophobicity.

The normalized regressed line (with zero intercept) can be obtained by substituting (6) into the original straight line equation yielding

(7) $$\begin{array}{c} \frac{x_{\Phi }-\mu_{\Phi }}{\sigma_{\Phi }}=\rho_{c,\Phi }\frac{x_{c}-u_{c}}{\sigma_{c}}\\ {\tilde{x}}_{\Phi }=\rho_{c,\Phi }\cdot {\tilde{x}}_{c} \end{array}$$

The normal to the regressed line at the origin where $\left({\tilde{x}}_{c},{\tilde{x}}_{\Phi }\right)=\left(0,0\right)$ is simply given as

(8) $${\tilde{x}}_{\Phi }=-\frac{1}{\rho_{c,\Phi }}\cdot {\tilde{x}}_{c}$$

### Computation of False-positive, False-negative, True-negative, False-discovery rates

For the purpose of benchmarking, the false-positive rate (FPR), false-negative rate (FNR) or (1-sensitivity) and false-discovery rate (FDR) are given as

(9) $$FPR=\frac{FP}{FP+TN}$$

(10) $$FNR=\frac{FN}{TP+FN}$$

(11) $$FDR=\frac{FP}{FP+TP}$$

respectively, where *FP, TN, TP, TN *are the total number of false-positives, true-negatives, true-positives and true-negatives in a dataset respectively.

## Competing interests

The authors declare that they have no competing interests.

## Authors' contributions

WCW, SMS and FE jointly conceived and designed the experiments. WCW and SMS performed the computations, WCW provided programs and scripts when not otherwise stated. WCW, SMS, and FE analyzed the data. WCW and FE drafted the manuscript text. All authors read and approved the final manuscript.

## Reviewers' Comments

### Reviewer's report 1

Shamil Sunyaev, Division of Genetics, Dept. of Medicine, Brigham & Women's Hospital and Harvard Medical School

This manuscript investigates differences in sequence complexity and hydrophobicity between transmembrane helices serving purely structural role and transmembrane helices with additional functional roles. On the practical side, the analysis of sequence complexity and hydrophobicity is able to identify transmembrane helices that are responsible for spurious sequence search hits as opposed to functional transmembrane helices that are useful for homology search. The manuscript is a follow up on an earlier manuscript by the same authors that suggested suppressing transmembrane segments in domain models to increase sensitivity and specificity of remote homology search. The results are of interest both in terms of protein evolution and in terms of practical utility for sequence similarity searches.

Authors' response

We thank the reviewer for highlighting these points. Even if the straightforward, silent extension of computing homology via sequence similarity from globular domains to membrane proteins is probably correct in a number of cases, it is important to identify this uncertainty as blind assumption and to derive criteria aimed at excluding situations when assigning homology in this simplified manner is not justified.

I have two minor comments:

1) Since sequence complexity and hydrophobicity seem to be correlated, I wonder why is not the inverse co-variance matrix included in the Z-score.

Authors' response

*If the distribution of the points *$\left({\tilde{x}}_{\Phi },{\tilde{x}}_{c}\right)$*(we use the normalized forms *${\tilde{x}}_{\Phi }=\left(x_{\Phi }-\mu_{\Phi }\right)∕\sigma_{\Phi }$*and *${\tilde{x}}_{c}=\left(x_{c}-\mu_{c}\right)∕\sigma_{c}$) *is considered in the hydrophobicity/complexity plot (Figure *3A), *we find that most of the points representing real TMs are either of high hydrophobicity/low complexity (simple TMs or of less hydrophobicity and higher complexity. The two alternative quadrants (low hydrophobicity/low complexity or high hydrophobicity/high complexity) are not much populated and, from the viewpoint of the concept developed in this article, it is not very clear how to deal with them meaningfully. Of course, one can develop analytically more sophisticated expressions for the Z-score as proposed by the reviewer and, maybe, it might prove useful in the future. Here, we wanted to have a very simple form of the Z-score that is symmetric across the four quadrants. At the end, we are only after TMs with low Z-score.*

2) On a more philosophical note, I would not state that "homology" equals to common evolutionary origin but rather than 'homology" is a surprisingly high level of similarity that cannot be explained by a functional constraint and, therefore, indicative of potentially common evolutionary origin.

Authors' response

We fully agree with the reviewer's view. The problem is in the practical detail: How do we know that there is no functional constraint for query sequences (whether there is convergent evolution or common ancestry)? From the sequence analytic perspective, homology is usually inferred from easily derived similarity measures of aligned sequences because there is no other direct measure of common ancestry, not because this criterion is especially elegant or rigorous. The practice becomes questionable when this caveat is forgotten.

### Reviewer's report 2

L. Aravind, Protein and Genome Evolution Research Group, Computational Biology Branch, NCBI/NLM/NIH

I entirely agree with the chief result presented in this article, namely: "Whereas simple TMs have the potential to confuse searches for sequence homologues and to generate unrelated hits with seemingly convincing statistical significance, complex TMs contain essential evolutionary information." This has been known from analysis of sequences of membrane proteins, particularly those associated with specific biochemical functions. The TM enzymes, channels and other unusual structures like the CYSTM or KASH, and to a lesser degree certain receptors like 7TM and 5TM receptors, indeed possess evolutionarily constrained features - resulting in what the authors term complex TMs. Hence, these tend not to "get corrupted" in sequence profile searches. The salient point of this paper is the quantitative treatment of this phenomenon - it would be of considerable value for those interested in the problems concerning homology in TM proteins.

Authors' response

We agree with the reviewer that the article provides a framework for deriving the respective quantitative criteria. We do not exclude that, in the future, modified forms of Z-score might prove even more efficient than the analytical forms provided in this work.

Some points the authors might want to consider: "It is possible that some of these simple helices harboring lysine are in fact complex."

This indeed seems to be the case. One might consider the example of conserved intra-TM lysines that are present in both in animal rhodopsins and bacteriorhodopsins, which appear to be complex 7 TM proteins (e.g. 10988064). This lysine is the target of covalent modification by the retinal prosthetic group of these proteins and is under evolutionary constraints. This seems to be a lysine that is distinct from the lysines that might play merely an anchoring role by interacting with negatively charged head groups of lipids. If the lysines in anchoring TMs are primarily linked to interactions with lipid head groups then would predict their occurrence closer to the ends of the helix.

"If this is indeed the case, then complex TM helices (implying common ancestry) do also exist in single-spanning membrane proteins, contrary to their role as only anchors (implying convergent evolution)."

One might consider the tail anchoring single TM like the CYSTM (19933165) which appears to be single complex TM family where a particular pattern of evolutionarily constrained cysteines point to a role beyond mere anchorage.

The APMAP TM segment: Based on purely evolutionary considerations I am not sure if this TM segment was always complex. Firstly, as the authors state this TM segment anchors the 6-bladed beta propeller strictosidine synthase-like domain (It is better not term it as a hydrolase as it catalyzes a Pictet-Spengler condensation along with the release of a water molecule rather than a genuine hydrolysis). The TM region, immediately N-terminal to the beta propeller is present in all orthologs of this group ranging from bacteria, plants and animals but is not conserved in sequence - an observation more consistent with its role as an anchor. It is possible that it acquired some additional constraints in vertebrates alone, but additional evidence in support of this would be needed to test its true complexity status.

Authors' response

We thank the reviewer for providing additional instructive examples of rhodopsins and CYSTM proteins. With regard to the APMAPs, we find in our analysis that the TM segment among the higher vertebrates (APMAP_CHICK, APMAP_RAT, APMAP_BOVIN, APMAP_HUMAN and APMAP_MOUSE) were either complex or in twilight range at z-scores of -1.74, -2.92, -3.76, -2.54 and -3.42 respectively. Most interestingly, we found that glycine and proline (conserved motif PLLGA) are conserved at the C-terminus while the aromatic residue, phenylalanine (conserved motif TFL) is conserved in the N-terminus on the TM segment. Given that these three residues were enriched in complex TMs in our findings, the TM segment in these vertebrates are likely more than just TM anchors and, possible, may have a role for intra-membrane oligomerization of TM regions from several proteins.

### Reviewer's report 3

Arcady Mushegian, Bioinformatics Center, Stowers Institute for Medical Research

The manuscript of Wong et al. presents good evidence that the transmembrane segments in proteins, which were thought to be compositionally biased and, because of this bias, not very useful in probabilistic sequence similarity searches, are in fact a heterogeneous group of sequences. Some TM segments are indeed "simple" and had better be masked before scanning the databases for homologs, whereas others are more complex, have family-specific distinguishing features and can be used for homology searches. These observations are useful and, I believe, largely correct, but I have three concerns.

First, I suggest a vigorous editing of all discussion of homology. It is not until the second paragraph on page 20 that the authors get to defining homology, and do it with a whiff of ambiguity: "Though homology has the precise meaning of "having a common evolutionary origin", it also carries the loose meaning of "possessing sequence similarity or being matched" when translated into computerized homology searches." I think that we should insist on the precise meaning of this word, and do not feed the troll of the loose meaning.

Authors' response

We share the concern of the reviewer that sequence homology should be used for denoting "having a common evolutionary origin". In the text, we describe how the term (and, more importantly, the concept) is used in sequence annotation exercises elsewhere, even in circumstances that are not well covered by empirical evidence such as SPs/TMs and other non-globular regions (in contrast to globular domains). Essentially, this article was initiated by the critical review of such cases in the literature/databases where the meaning of homology was watered down.

*'Biological homology' has a broad definition among biologists from paleontologist to evolutionists *[67]. *A computable definition of 'homology' that is all encompassing is an unsolved, formidable task. This practical inconvenience is the driving force behind determining sequence homology via sequence similarity; yet, this proxy to hypothesize homology is only sensible as long as the researchers are aware about the validity restrictions of this criterion.*

Homology exists regardless of our ability to detect it (and this ability, by the way, improves as we have more data and come up with the new ideas). Thus, in a sharp contrast with what the title of the paper misleadingly says, there is no need to "extend the NOTION of homology to transmembrane proteins" --this notion already covers all types of proteins.

Authors' response

We do agree that homology exists regardless of our ability to detect it and it is not our intent to create a novel concept that membrane proteins can be homologous as well. However, we wish to provide arguments to distinguish whether the traditional approach of determining homology via sequence similarity is appropriately applied to given TM proteins and TM regions. The title aims to explicitly caution the general reader against the direct application of the sequence similarity criterion for homology of membrane proteins given the heterogeneity of TM helices.

What may be needed is the improvement of our skills in distinguishing common ancestry from convergent or parallel evolution in the case of compositionally biased proteins.

Authors' response

Indeed, an improvement in distinguishing convergent evolution and common ancestry for compositionally biased proteins is necessary. In this case, we formulated the necessary z-score criteria to distinguish simple (implies possible convergent evolution) from complex (implies likely common ancestry) TMs.

This brings me to the second concern, and it is that the authors leave the reader without any practical guidance. Yes, we are told to mask simple TMs and not to mask complex TMs, but is there a program or a server that would help one to do it? Can one collect the TM segments using their favorite prediction engine and run these segments through an R script, for example?

Authors' response

*In the mean time, we have a program source ready for distribution to the community available from the WWW site associated with this article (*http://mendel.bii.a-star.edu.sg/SEQUENCES/ProblemDomains-TM-classification/)*. The program is written as PERL modules and requires the TM region(s) and input sequence to be defined as inputs.*

Speaking of this, are the efforts from Altschul and Yu groups at NCBI on compositional bias and adjustment of scoring matrices of any relevance. Will these approaches treat different kinds of TM segments appropriately?

Authors' response

Indeed, there are two complementary approaches to deal with compositional bias in sequences. One relies of score scaling computed from the queries and/or the database sequences' amino acid compositions. There are several flavors of compositional bias correction described in the literature; it is out of the scope of this MS to evaluate their performance on regions that have the compositional bias of a simple TM.

*The alternative is suppressing/excluding compositionally biased regions using prior information. The filtering of query sequences by sequence complexity computation programs such as SEG have a long history in sequence similarity searches. Less systematically, other non-globular segments such as coiled coils are excluded from similarity searches *[11]. *As a result of this work, we suggest to determine hydrophobicity and sequence complexity of TM regions and to suppress those with low Z-scores (simple TMs). To note, SEG does not cover most simple TMs as shown in this work.*

It appears to us that both approaches have their right for existence. And they have their own advantages and deficiencies. Whereas the compositional bias score corrections are applicable for all types of sequences, they run into difficulties over which part of the sequence to determine the amino acid composition (and using the whole sequence is not a good choice in multi-segment proteins). Usually, using prior knowledge is more robust for suppressing compositionally biased segments; yet, this is less convenient if existing software pipelines do not include all programs required.

My third concern is the manuscript style, reminiscent of a personal memoir or an oral presentation. I do believe that the quirks and idiosyncrasies of style have their place in scientific literature, but in this case the text is way too verbose. I suggest that the authors revise the whole manuscript and suspect that the main text and Figure Legends may become twice as short as they are now, to the benefit of the reader.

Several random examples, in no way exhaustive:

p. 2: < <The work presented in this article has essentially emerged in response to an important comment by one of the reviewers of our previous publications [1]; namely, the complete exclusion of TMs from domain models in libraries such as Pfam [12,13] "would be a huge disservice to the community" and certain domain models involving TM regions have proven instrumental in protein family classification as, for example, in the cataloging of membrane transporters by Saier and coworkers [14-16].> >

--consider replacing everything before "certain domain models" by "On the other hand,"

p.3: "For sequence similarity applications within the sequence homology concept (i.e., for the extension of the homology concept to membrane proteins), a quantitative criterion for distinction between complex and simple TMs would be very helpful, not only in the context of automated annotation pipelines."--consider deleting completely and see whether the logic of the narrative suffers at all.

p.4: "Figure 1A shows a three-dimensional fin-shaped histogram of all TM helices spanning diagonally across the sequence complexity and hydrophobicity space. The cross-section of the skewed histogram (Figure 1B) shows a tear shaped scatter plot."

--describes exactly what is in the figure; appropriate in an oral presentation, not so much in a written article.

In the Legend to Figure 1, the exact same sentences are repeated, and more along the same lines is seen, e.g. "The trace of the complexity and hydrophobicity from the single-spanning set to the four-TM spanning set indicates a progressive shift towards high complexity and low hydrophobicity. The medians converge to almost a singular cloud beyond the five-transmembrane-spanning set."

--again, this is a literal description of what is in the figure -redundant!

p. 5: "The data shows that there are a sizeable number of outliers not following the trends and we can reconcile this seemingly contradictory observation by acknowledging that simple and complex TMs can occur in proteins regardless of the total number of TMs."

--consider replacing with "The data suggests that simple and complex TMs can occur in proteins regardless of the total number of TMs."

p. 7:"Our general observation that complex TM helices are enriched with charged and structural residues" --tautology? "Complex TM" is by definition a TM that is enriched in these residues, no?

Authors' response

We thank the reviewer for his suggestions and for selecting some examples. We carefully re-examined the text of the article and modified/shortened some segments including some of those listed by the reviewer. At the same time, we wish to emphasize that we are dissatisfied with the trend to a telegram-style writing in scientific publications. Just showing a graph does not necessarily evoke the same thoughts in the minds of readers from diverse backgrounds and, to some, the conclusion chain might not be clear. Therefore, it makes sense to describe in words what the authors think to see. Similarly, one and the same wording can be associated with different meanings in the understanding of various researchers," sequence homology" being a prominent example. Thus, it is not superfluous to emphasize that we have common evolutionary origin in mind and that sequence similarity is only an imperfect means for its detection. To conclude, we wish to provide utmost clarity in methodology and conclusion chains and, at the same time, some entertaining reading if possible given the limitations due to the heavy technicality of this work.

## Supplementary Material

Additional file 1 **Supplementary Table 1**. This table contains the statistically-enriched residues in the anchor sets (membrane/signal) and functional TM-helix sets (UniProt/SCOP-derived) when tested against each other through 2-by-2 contingency tables. Click here for file

Additional file 2 **Supplementary Table 2**. This table contains the correlation values between the four sets of statistically-enriched residues derived from Additional file 1, Supplementary Table 1 and a set of experimentally-derived hydrophobicity scale. Click here for file
