# Supplementary material for: Not all transmembrane helices are born equal: Towards the extension of the sequence homology concept to membrane proteins
Source: Biol Direct. 2011 Oct 25;6:57. doi: 10.1186/1745-6150-6-57 (PMC3217874; doi:10.1186/1745-6150-6-57)
Supplement: Additional file 1 — Supplementary Table 1. This table contains the statistically-enriched residues in the anchor sets (membrane/signal) and functional TM-helix sets (UniProt/SCOP-derived) when tested against each other through 2-by-2 contingency tables. [file 1745-6150-6-57-S1.PDF]

Supplementary Table 1a. **Membrane anchors versus functional TMs (SCOP-derived)**

Residue	H_o: p1=p2	Chi-square, χ^2	Significance at p ≤ 0.0025	Sign
A	0.095; 0.114	5.55	no	+
R	0.007; 0.017	12.12	yes	+
N	0.005; 0.015	21.42	yes	+
D	0.002; 0.007	11.08	yes	+
C	0.026; 0.012	12.70	yes	-
E	0.001; 0.009	35.78	yes	+
Q	0.006; 0.011	5.08	no	+
G	0.059; 0.095	28.96	yes	+
H	0.005; 0.048	181.81	yes	+
I	0.173; 0.093	72.35	yes	-
L	0.220; 0.136	64.77	yes	-
K	0.013; 0.007	3.89	no	-
M	0.037; 0.053	8.92	no	+
F	0.082; 0.111	14.47	yes	+
P	0.013; 0.030	25.62	yes	+
S	0.035; 0.040	0.98	no	+
T	0.034; 0.043	3.34	no	+
W	0.023; 0.032	4.72	no	+
Y	0.027; 0.033	2.36	no	+
V	0.138; 0.094	24.77	yes	-

Supplementary Table 1b. **Membrane anchors versus functional TMs (UnitProt-derived)**

Residue	H_o:p1=p2	Chi-square, χ^2	Significance at p ≤ 0.0025	Sign
A	0.095; 0.108	8.68	no	+
R	0.007; 0.016	25.46	yes	+
N	0.005; 0.019	61.59	yes	+
D	0.002; 0.014	63.79	yes	+
C	0.026; 0.017	26.58	yes	-
E	0.001; 0.006	27.66	yes	+
Q	0.006; 0.007	2.01	no	+
G	0.059; 0.087	53.22	yes	+
H	0.005; 0.047	243.22	yes	+
I	0.173; 0.109	216.40	yes	-
L	0.220; 0.160	144.83	yes	-
K	0.013; 0.003	128.39	yes	-
M	0.037; 0.039	0.68	no	+
F	0.082; 0.123	86.26	yes	+
P	0.013; 0.026	36.21	yes	+
S	0.035; 0.040	4.07	no	+
T	0.034; 0.037	2.06	no	+
W	0.023; 0.025	0.38	no	+
Y	0.027; 0.033	6.80	no	+
V	0.138; 0.088	154.11	yes	-

Supplementary Table 1c. **Signal anchors versus functional TMs (SCOP-derived)**

Residue	H_o: p1=p2	Chi-square, χ^2	Significance at p ≤ 0.0025	Sign
A	0.113; 0.114	0.00	no	+
R	0.008; 0.017	14.34	yes	+
N	0.008; 0.015	10.09	yes	+
D	0.002; 0.008	15.29	yes	+
C	0.029; 0.012	19.79	yes	-
E	0.003; 0.009	19.39	yes	+
Q	0.009; 0.011	0.51	no	+
G	0.066; 0.095	23.91	yes	+
H	0.005; 0.048	449.32	yes	+
I	0.101; 0.093	1.06	no	-
L	0.244; 0.136	116.75	yes	-
K	0.008; 0.007	0.00	no	-
M	0.025; 0.053	54.73	yes	+
F	0.083; 0.111	17.37	yes	+
P	0.015; 0.030	25.52	yes	+
S	0.054; 0.040	7.13	no	-
T	0.046; 0.043	0.39	no	-
W	0.020; 0.032	14.25	yes	+
Y	0.032; 0.033	0.07	no	+
V	0.129; 0.094	19.41	yes	-

Supplementary Table 1d. **Signal anchors versus functional TMs (UnitProt-derived)**

Residue	H_o: p1=p2	Chi-square, χ^2	Significance at p ≤ 0.0025	Sign
A	0.113; 0.108	5.58	no	-
R	0.008; 0.016	89.38	yes	+
N	0.008; 0.019	145.97	yes	+
D	0.002; 0.014	314.28	yes	+
C	0.029; 0.017	135.22	yes	-
E	0.003; 0.006	35.96	yes	+
Q	0.009; 0.007	5.72	no	-
G	0.066; 0.087	113.71	yes	+
H	0.005; 0.047	1270.54	yes	+
I	0.101; 0.109	12.87	yes	+
L	0.244; 0.159	829.41	yes	-
K	0.008; 0.003	80.58	yes	-
M	0.025; 0.039	124.25	yes	+
F	0.083; 0.123	313.06	yes	+
P	0.015; 0.026	96.72	yes	+
S	0.054; 0.040	80.39	yes	-
T	0.046; 0.037	34.93	yes	-
W	0.020; 0.025	20.22	yes	+
Y	0.032; 0.033	0.29	no	+
V	0.129; 0.088	316.84	yes	-

For each table, the first column contains the amino acid residue type. The second column gives the null hypothesis for the test of proportion. p1 and p2 listed here measure the proportion of the particular residue in the anchor populations (membrane/signal) and functional TM-helices (SCOP-/UniProt-derived) respectively. The third column contains the chi-square χ^2 values that are derived from 2-by-2 contingency table (particularly the binomial comparative trial) for each AA alphabet (see J.H.Zar, Biostatistical analysis, chapter 23, pp. 491-500). The forth column gives the significance state (“yes” or “no”) of the statistical test for a given significant level. The family-wise error rate for the 20 tests (given 20 residues in each population) is set at p=0.05, hence each test is set at p ≤ 0.0025 (i.e. 0.05 ÷ 20). The last column denotes the directionality of the proportion p1 and p2 (i.e. “– “ if p1>p2 and “+” if p1<p2).

The residues that are enriched in either set are marked with color shading (red for the anchor TM set and blue for the functional TM set). In Table 1d, isoleucine was excluded from the list of marked amino acid types since it only marginally matched the significance test.
