# Supplementary material for: Not all transmembrane helices are born equal: Towards the extension of the sequence homology concept to membrane proteins
Source: Biol Direct. 2011 Oct 25;6:57. doi: 10.1186/1745-6150-6-57 (PMC3217874; doi:10.1186/1745-6150-6-57)
Supplement: Additional file 2 — Supplementary Table 2. This table contains the correlation values between the four sets of statistically-enriched residues derived from Additional file 1, Supplementary Table 1 and a set of experimentally-derived hydrophobicity scale. [file 1745-6150-6-57-S2.PDF]

Supplementary Table 2a. **Correlation between the hydrophobicity scale and Chi-square value of significantly enriched residues between the membrane anchors and functional TMs (SCOP-derived)**

Residue	Hydrophobicity scale	χ^2 x sign
R	1.00	12.12
N	0.43	21.42
D	2.41	11.08
C	0.22	-12.70
E	1.61	35.78
G	1.14	28.96
H	1.37	181.81
I	-0.81	-72.35
L	-0.69	-64.77
F	-0.58	14.47
P	-0.31	25.62
V	-0.53	-24.77
Correlation, ρ = 0.56		

Supplementary Table 2b. **Correlation between the hydrophobicity scale and Chi-square value of significantly enriched residues between the membrane anchors and functional TMs (UniProt-derived)**

Residue	Hydrophobicity scale	χ^2 x sign
R	1.00	25.46
N	0.43	61.59
D	2.41	63.79
C	0.22	-26.58
E	1.61	27.66
G	1.14	53.22
H	1.37	243.22
I	-0.81	-216.40
L	-0.69	-144.83
K	1.81	-128.39
F	-0.58	86.26
P	-0.31	36.21
V	-0.53	-154.11
Correlation, ρ = 0.46		

<u>Supplementary Table 2c.</u> Correlation between the hydrophobicity scale and Chi-square value of significantly enriched residues between the signal anchors and functional TMs (SCOP-derived)

Residue	Hydrophobicity scale	χ^2 x sign
R	1.00	14.34
N	0.43	10.09
D	2.41	15.29
C	0.22	-19.79
E	1.61	19.39
G	1.14	23.91
H	1.37	449.32
L	-0.69	-116.75
M	-0.44	54.73
F	-0.58	17.37
P	-0.31	25.52
W	-0.24	14.25
V	-0.53	-19.41
Correlation, ρ = 0.36		

<u>Supplementary Table 2d.</u> Correlation between the hydrophobicity scale and Chi-square value of significantly enriched residues between the signal anchors and functional TMs (UniProt-derived)

Residue	Hydrophobicity scale	χ^2 x sign
R	1.00	89.38
N	0.43	145.97
D	2.41	314.28
C	0.22	-135.22
E	1.61	35.96
G	1.14	113.71
H	1.37	1270.54
I	-0.81	12.87
L	-0.69	-829.41
K	1.81	-80.58
M	-0.44	124.25
F	-0.58	313.06
P	-0.31	96.72
S	0.33	-80.39
T	0.11	-34.93
W	-0.24	20.22
V	-0.53	-316.84
Correlation, ρ = 0.42		

For each table, the first column contains the amino acid residues that are found to be significantly enriched based on its corresponding supplementary tables 1. The second column gives the respective hydrophobicity scales. These scale values were extracted from the Octanol-interface scale of “White SH, Wimley WC (1998) Hydrophobic interactions of peptides with membrane interfaces. Biochim Biophys Acta 1376: 339-352”. The last column contains

the product of the chi-square χ^2 and sign from column 3 and 5 of corresponding supplementary tables 1. Since χ^2 values cannot be negative, the sign gives the directionality to the final product based on the proportion inequality “p1>p2” or “p1<p2” in order to complement the directionality of the hydrophobicity scale. The last row gives the correlation between column 2 (the hydrophobicity scale) and 3 (product of χ^2 and sign).
